# C_4_ gene induction during de-etiolation evolved through changes in cis to allow integration with ancestral C_3_ gene regulatory networks

**DOI:** 10.1126/sciadv.ade9756

**Published:** 2023-03-29

**Authors:** Pallavi Singh, Sean R. Stevenson, Patrick J. Dickinson, Ivan Reyna-Llorens, Anoop Tripathi, Gregory Reeves, Tina B. Schreier, Julian M. Hibberd

**Affiliations:** Department of Plant Sciences, University of Cambridge, Downing Street, Cambridge CB2 3EA, UK.

## Abstract

C_4_ photosynthesis has evolved by repurposing enzymes found in C_3_ plants. Compared with the ancestral C_3_ state, accumulation of C_4_ cycle proteins is enhanced. We used de-etiolation of C_4_ *Gynandropsis gynandra* and C_3_ *Arabidopsis thaliana* to understand this process. C_4_ gene expression and chloroplast biogenesis in *G. gynandra* were tightly coordinated. Although C_3_ and C_4_ photosynthesis genes showed similar induction patterns, in *G. gynandra*, C_4_ genes were more strongly induced than orthologs from *A. thaliana*. In vivo binding of TGA and homeodomain as well as light-responsive elements such as G- and I-box motifs were associated with the rapid increase in transcripts of C_4_ genes. Deletion analysis confirmed that regions containing G- and I-boxes were necessary for high expression. The data support a model in which accumulation of transcripts derived from C_4_ photosynthesis genes in C_4_ leaves is enhanced because modifications in cis allowed integration into ancestral transcriptional networks.

## Teaser

C_4_ photosynthesis evolved through the acquisition of light responsive elements in the regulatory regions of C_4_ genes.

## INTRODUCTION

Photosynthesis fuels most life on Earth, and, in most land plants, ribulose-1,5-bisphosphate carboxylase-oxygenase (RuBisCO) catalyzes the initial fixation of atmospheric carbon dioxide to generate phosphoglyceric acid. However, oxygen can competitively bind the RuBisCO active site and, in doing so, form the toxic product 2-phosphoglycolate (*1*). Although 2-phosphoglycolate can be metabolized by photorespiration (*2*), this takes place at the expense of carbon and energy, and it is thought that the evolution of carbon concentrating mechanisms allowed rates of photorespiration to be reduced.

The C_4_ pathway is one such example and characterized by the partitioning of photosynthesis into two compartments: typically, mesophyll and bundle sheath cells (*3*). This compartmentation involves cell-preferential gene expression and allows increased concentrations of CO_2_ to be supplied to RuBisCO, which becomes restricted to bundle sheath cells (*4*). Thus, rather than RuBisCO initially fixing carbon, in C_4_ species, fixation is initiated by phosphoenolpyruvate carboxylase combining HCO_3_^−^ to form a C_4_ acid in the mesophyll. Diffusion of C_4_ acids into bundle sheath cells and subsequent decarboxylation results in elevated partial pressures of CO_2_ around RuBisCO, facilitating efficient carboxylation and reducing the requirement for substantial rates of photorespiration. C_4_ photosynthesis results in higher water and nitrogen use efficiencies compared with the C_3_ state, particularly in dry and hot climates. C_4_ crops of major economic importance include maize (*Zea mays*), sugarcane (*Saccharum officinarum*), sorghum (*Sorghum bicolor*), pearl millet (*Pennisetum glaucum*), and finger millet (*Setaria italica*) (*5*). Although C_4_ photosynthesis is a complex trait characterized by changes in anatomy, biochemistry, and gene expression (*3*), it has evolved convergently from C_3_ ancestors in more than 60 independent lineages that together account for ~8100 species (*5*). Parsimony would imply that gene networks underpinning this system are derived from those that operate in C_3_ ancestors.

Compared with the ancestral C_3_ state, expression of genes encoding components of the C_4_ pathway are up-regulated and restricted to either mesophyll or bundle sheath cells. Our present understanding of these changes to C_4_ gene regulation is mostly based on studies designed to understand the regulation of individual C_4_ genes (*6*). For example, a number of cis-regulatory motifs controlling the cell preferential expression of C_4_ genes have been identified (*7*–*12*). While some cis-elements appear to have evolved de novo in C_4_ genes to pattern their expression (*13*–*16*), others appear to have been recruited from preexisting sequence present in C_3_ orthologs (*9*–*12*), and, in these cases, there is evidence that individual cis-elements are shared between multiple C_4_ genes.

In contrast to the analysis of regulators of cell-specific expression, there has been very little work designed to understand mechanisms that underpin the up-regulation and response to light of C_4_ genes. For example, while many C_4_ pathway components and their orthologs in C_3_ species show light-dependent induction (*17*), the mechanisms driving these patterns are unknown. One possibility is that cis-elements referred to as light-responsive elements (LREs) that have been characterized in photosynthesis genes in C_3_ plants (*18*) are acquired by genes of the core C_4_ pathway. The response of a seedling to light is the first major step toward photosynthetic maturity, but how C_4_ genes are integrated into this process is poorly understood. Seedlings exposed to prolonged darkness develop etioplasts in place of chloroplasts (*19*). Etioplasts lack chlorophyll but contain membranes composed of a paracrystalline lipid-pigment-protein structure known as the prolamellar body (*20*). De-etiolation of seedlings therefore marks the initiation of photosynthesis and presents a good model system to study the dynamics and regulatory mechanisms governing photosynthesis in both C_3_ and C_4_ species.

Here, we used an unbiased large-scale genome-wide approach to better understand the patterns of transcript abundance and potential regulatory mechanisms responsible for the induction of C_4_ gene expression. This is the first attempt to integrate chromatin accessibility, predicted transcription factor binding sites, as well as binding evidence obtained in vivo and expression data to understand the regulation of gene expression in the C_4_ species *Gynandropsis gynandra*. In particular, we focus on how core C_4_ pathway genes are strongly induced and reach high transcript abundances in relation to their orthologs in *Arabidopsis thaliana*. Reanalysis of a publicly available dataset from *A. thaliana* (*21*) allowed us to compare the extent to which regulatory mechanisms are shared between the ancestral C_3_ and derived C_4_ systems. This direct comparison between the derived C_4_ and the ancestral C_3_ photosynthetic state provided insights into the evolution of C_4_ photosynthetic traits. We identified not only cis-elements that were enriched in regions of C_4_ genes targeted by transcription factors to regulate gene expression, hereafter referred to as the C_4_ cistrome, including those related to TGACG-binding factor (TGA), teosinte branched1/cincinnata/proliferating cell factor (TCP), and homeodomain (HD) transcription factor binding, but also G-box motifs that were enriched in both C_3_ and C_4_ cistromes. Analysis of transcription factor binding in vivo suggested an association between the presence of TGA, HD motifs, and LREs such as G- and I-boxes and strong induction during de-etiolation. Different C_4_ genes in *G. gynandra* contain distinct sets of LREs, but promoter deletion analysis of the *pyrophosphorylase 6* (*PPa6*) gene showed that regions containing G- and I-boxes were necessary for high expression. Together, these data present compelling evidence that C_4_ genes are rewired to allow integration into existing gene regulatory networks to permit rapid, robust, and spatially precise induction of C_4_ gene expression during de-etiolation.

## RESULTS

### De-etiolation and chloroplast development in C_4_ *G. gynandra*

**Fig. 1.**
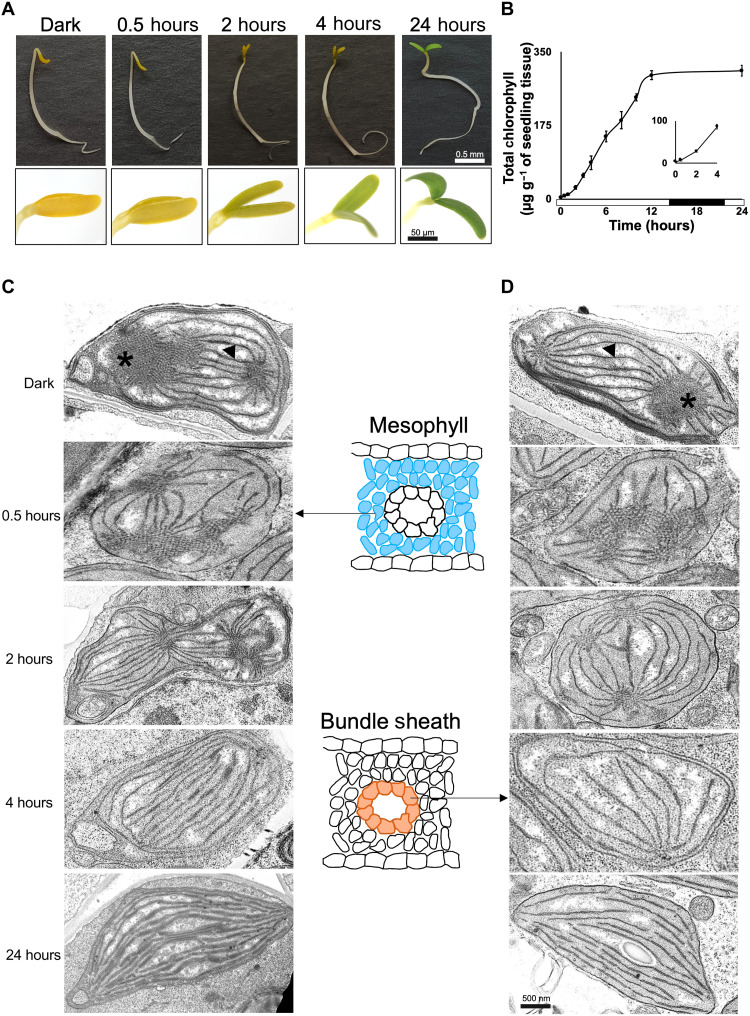
Response of cotyledons to light in *G. gynandra*. (**A**) Representative images of *G. gynandra* seedlings illustrating greening and unhooking of the cotyledons. (**B**) Total chlorophyll over time (data shown as means from three biological replicates at each time point, ± 1 SD from the mean). First 4 hours shown in inset. Bar along the *x* axis indicates periods of light (0 to 14 and 22 to 24 hours) or dark (14 to 22 hours). (**C** and **D**) Representative transmission electron micrographs of etioplast to chloroplast transition in mesophyll (C) and bundle sheath cells (D) at 0, 0.5, 2, 4, and 24 hours after exposure to light. Asterisks and arrowheads indicate the prolamellar body and prothylakoid membranes, respectively. Scale bars represent 0.5 mm for seedlings (A) and 50 μm for cotyledons (A) and 500 nm (C and D).

We determined the dynamics of unfolding of the apical hook, chlorophyll accumulation, and ultrastructural rearrangements of chloroplasts as etiolated seedlings of *G. gynandra* were exposed to light. The classical photomorphogenic responses of apical hook unfolding and greening of cotyledons were visible 2 hours after transfer to light (Fig. 1A). Chlorophyll accumulation was detectable by 0.5 hours after exposure to light, and an initial exponential phase was followed by a more linear increase (Fig. 1B). Little additional chlorophyll was synthesized from 12 to 24 hours when plants were in the dark (Fig. 1B). Assembly of the photosynthetic membranes in chloroplasts from mesophyll and bundle sheath cells, both of which are involved in C_4_ photosynthesis, was apparent over this time course (Fig. 1, C to D). In the dark, prolamellar bodies dominated the internal space of chloroplasts in each cell type. After 0.5 hours of light, prolamellar bodies had started to disperse. Starch granules were detected in bundle sheath chloroplasts by 24 hours after exposure to light, and it was noticeable that thylakoids showed low stacking in this cell type (Fig. 1, C and D, and fig. S1). Overall, these data indicate that, in *G. gynandra*, assembly of the photosynthetic apparatus was initiated within 0.5 hours of exposure to light, and, by 24 hours, the apparatus appeared fully functional. To better understand the patterns of gene expression and the transcriptional regulation that underpin this induction of C_4_ photosynthesis, these early time points were selected for detailed molecular analysis.

### Induction of photosynthesis genes in *G. gynandra*

**Fig. 2.**
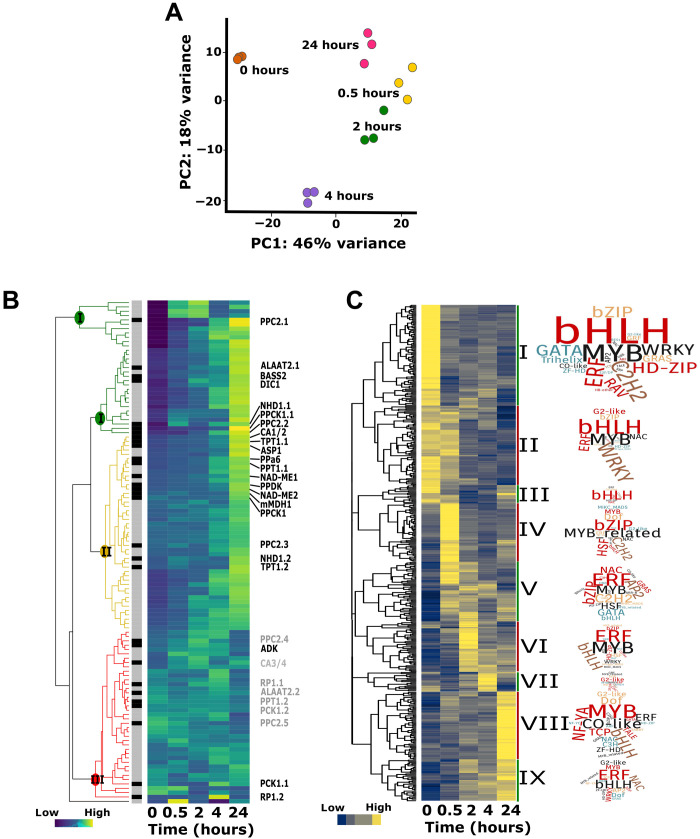
Changes in transcript abundance during greening of *G. gynandra*. (**A**) Principal components analysis of RNA-seq data. Biological replicates from each time point formed distinct clusters. PC, principal component. (**B**) Heatmap illustrating changes in transcript abundance of photosynthesis (gray sidebar) and C_4_ photosynthesis genes (black sidebar) during the time course. Color coding of the dendrograms (green, yellow, and red) highlight expression clusters representing strong, moderate, and no induction, respectively. Data shown after normalization of expression data with each gene plotted on a row and centered around the row mean. (**C**) Heatmap showing nine clusters containing 434 differentially expressed transcription factors. Values shown as *z* scores with yellow and blue indicating higher and lower values, respectively. Transcription factor families associated with each cluster are depicted in word clouds.

To investigate how transcript abundance changed during the induction of C_4_ photosynthesis, mRNA from three biological replicates at 0, 0.5, 2, 4, and 24 hours after exposure to light was isolated and used for RNA sequencing (RNA-seq). On average, 10 million reads were obtained, and ~25,000 transcripts detected per sample (table S1). Principal components analysis indicated replicates from each time point clustered together tightly, and that 64% of the variance in transcript abundance could be explained by two components (Fig. 2A). The first accounted for 46% of the variance and was primarily associated with the dark-to-light transition, while the second accounted for 18% and was linked to time after transfer to light.

Having established consistency between biological replicates, we next sought to better understand the behavior of photosynthesis genes, i.e., those that code for components of the photosynthetic apparatus such as *psbA* and *rbcL* (table S2), by subjecting them to hierarchical clustering (Fig. 2B). The 116 genes in this group separated into three broad behaviors corresponding to strong (cluster I, green), moderate (cluster II, yellow), and no clear induction (cluster III, red). Clusters I and II contained genes encoding enzymes involved in C_4_ photosynthesis (hereafter, C_4_ genes; table S2), and while this also appeared to be the case for cluster III, further investigation showed that these were poorly expressed paralogs of strongly induced C_4_ genes in cluster I or II.

To provide insight into the dynamics of gene expression during de-etiolation, we next analyzed how transcript abundance changed relative to each previous time point. Two independent algorithms were used, and the intersect between these datasets was used to obtain a conservative estimate of the number of differentially expressed genes (DEGs) between consecutive time points (table S1). The greatest difference in transcript abundance was detected 0.5 hours after transfer from dark to light (DEGs, 4609) with subsequent time points showing less than half of this number (table S1). To provide insight into the types of genes that were responding, Gene Ontology (GO) terms were assessed (fig. S2), and this showed that up-regulated genes at 0.5 and 24 hours showed enrichment not only in terms related to the plastid, as well as carbohydrate, secondary, nitrogen, and lipid metabolism, but also in responses to light and photosynthesis (fig. S2).

In addition to groups of genes relating to assembly of the photosynthetic apparatus in C_4_ leaves, genes that responded within 0.5 hours of light induction also included many transcription factors. To provide further insights into this, we subjected 434 transcription factors that were differentially expressed between at least two consecutive time points to hierarchical clustering (Fig. 2C and table S3). This identified nine clusters with differing peaks in expression across the time course. Clusters I to III represented around a third of the transcription factors that were down-regulated in response to light and contained many basic helix-loop-helix (bHLH) transcription factors (Fig. 2C). This also included BIRD/INDETERMINATE DOMAIN (IDD) and GIBBERELLIN INSENSITIVE (GAI), REPRESSOR of ga1-3 (RGA), and SCARECROW (SCR) transcription factors that are thought to play roles in regulating cellular patterning as well as many HD-Zip and zinc finger HD factors (*22*). In contrast, clusters IV and V showed rapid induction in response to light. Cluster IV contained many basic leucine zipper (bZIP), heat shock factors, and myeloblastosis (MYB)–related transcription factors that peaked after 0.5 hours of light, while cluster V included orthologs of well-known regulators of chloroplast development such as GATA nitrate-inducible carbon-metabolism-involved (GNC) and golden-like (GLK), as well as the master regulator of photomorphogenesis such as elongated hypocotyl 5 (HY5). The rapid up-regulation of transcription factors associated with cellular patterning followed by those associated with greening and chloroplast biogenesis is therefore consistent with previous analysis of C_3_ species.

### Chromatin dynamics associated with de-etiolation in *G. gynandra*

**Fig. 3.**
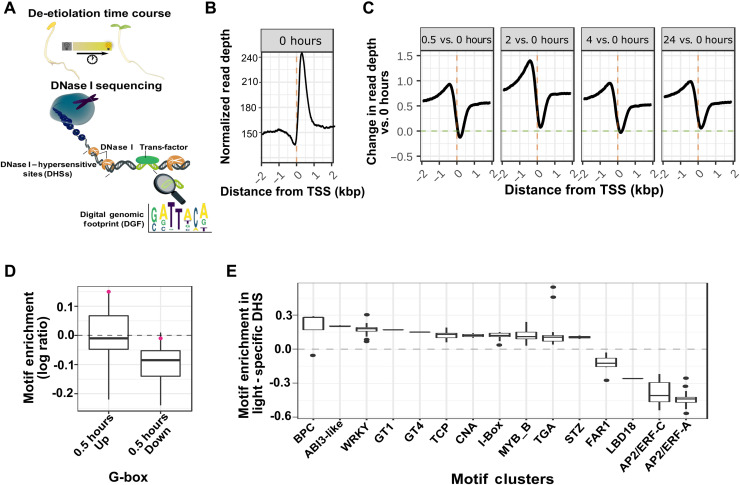
Chromatin profiling during de-etiolation of *G. gynandra*. (**A**) Schematic illustrating DNase I–seq pipeline including identification of DNase I–hypersensitive sites (DHSs). (**B**) Average normalized read depth around genes at 0 hours (dark). Predicted transcription start sites (TSSs) designated with vertical dashed orange line. kbp, kilo–base pair. (**C**) Average changes (log ratio) in normalized read counts around TSS for light time points (0.5, 2, 4, and 24) compared with the dark. Horizontal dashed line shows log ratio of 0 representing no change from dark, and vertical dashed line shows the predicted TSS. (**D**) At 0.5 hours, G-box motifs were enriched in genes up-regulated in response to light and depleted in genes repressed by light. *Elongated hypocotyl 5* (*HY5*) is indicated by the red dot. (**E**) Enriched and depleted motif clusters associated with light-specific DHSs.

We next used deoxyribonuclease I sequencing (DNaseI–seq) (Fig. 3A) to initiate an understanding of how chromatin accessibility might affect on the changes in gene expression described above. DNA was isolated from triplicate samples of nuclei and based on preliminary quality control (QC) metrics, one replicate from each time point was selected for deep sequencing (*21*). Raw reads were filtered, and, from the 15 samples, 1,145,530,978 mapped to the genome. In the dark, fewer reads mapped to the proximal promoter, and most reads mapped downstream of the transcription start site (TSS) (Fig. 3B). Notably, after exposure to light, this pattern altered such that sequences upstream and downstream of the TSS became more and less accessible, respectively (Fig. 3C). Increased accessibility upstream of the TSS was most noticeable 2 hours after exposure to light.

To limit the chances of false positives and to increase the chances of identifying high-confidence regions of open chromatin, only DNase I–hypersensitive sites (DHSs) detected in all five time points were selected. To do this, two shallow replicates for each time point were pooled and compared with each deep sequenced replicate (table S4). The DHSs were identified from the shallow and deep sequenced samples overlapped more than would be expected by chance, and intersection of these data identified 36,335 DHS found at all five time points. Hereafter, these are referred to as hyperconserved DHS. A further 4375 DHSs were present in the four time points from the light but were absent in the dark. We searched this combined list of 40,710 DHSs for cis-elements associated with the response of gene expression to light. To identify motifs specifically associated with genes that responded to light rather than the light-responsive DHS, we next selected DHS from all genes that were significantly up- or down-regulated at 0.5 hours and assessed their motif content. Notably, genes up-regulated at 0.5 hours contained significantly more G-box motifs than those that were down-regulated (Fig. 3D), and the most strongly enriched member of this motif group was that of HY5. As many DNA motifs are similar to each other, we collapsed 524 previously defined motifs (*23*) into 49 groups that typically correspond to particular families of transcription factors (table S5) and determined whether any such motif groups were overrepresented in the set of 4375 DHSs that responded to light. This approach showed that basic pentacysteine (BPC), WRKY, TCP, I-box, MYB, and TGA/bZIP motifs were enriched in the light-specific DHS, while two groups of APETALA2/ETHYLENE RESPONSIVE FACTOR (AP2/ERF) motifs were strongly depleted (Fig. 3E). In summary, these data indicate genome-wide reorganization of chromatin with proximal promoter regions showing increased accessibility in the light. Around 4000 conserved DHSs were detected in all time points after light exposure, and DHSs associated with genes up-regulated specifically at 0.5 hours were overrepresented with motifs associated with the response of gene expression to light.

### A cis-regulatory atlas for de-etiolation in *G. gynandra*

The chromatin accessibility assays and in silico analysis above identified regulatory elements that could be important for gene regulation but cannot indicate whether motifs were actually bound by transcription factors. We therefore carried out sequencing at sufficient depth to define DNA sequences protected from DNase I digestion. Such sequences are diagnostic of strong and/or widespread protein binding and referred to as digital genomic footprints (DGFs). To minimize false positives, such DGFs were defined only within the ~40,000 conserved DHSs identified above. The DNase I enzyme has sequence bias, and, to account for this, deproteinated DNA was analyzed to enable footprint filtering (*24*, *25*). After such filtering, 263,404 total and 193,143 unique (nonoverlapping) DGFs corresponding to individual transcription factor binding sites across all time points were predicted (fig. S3).

**Fig. 4.**
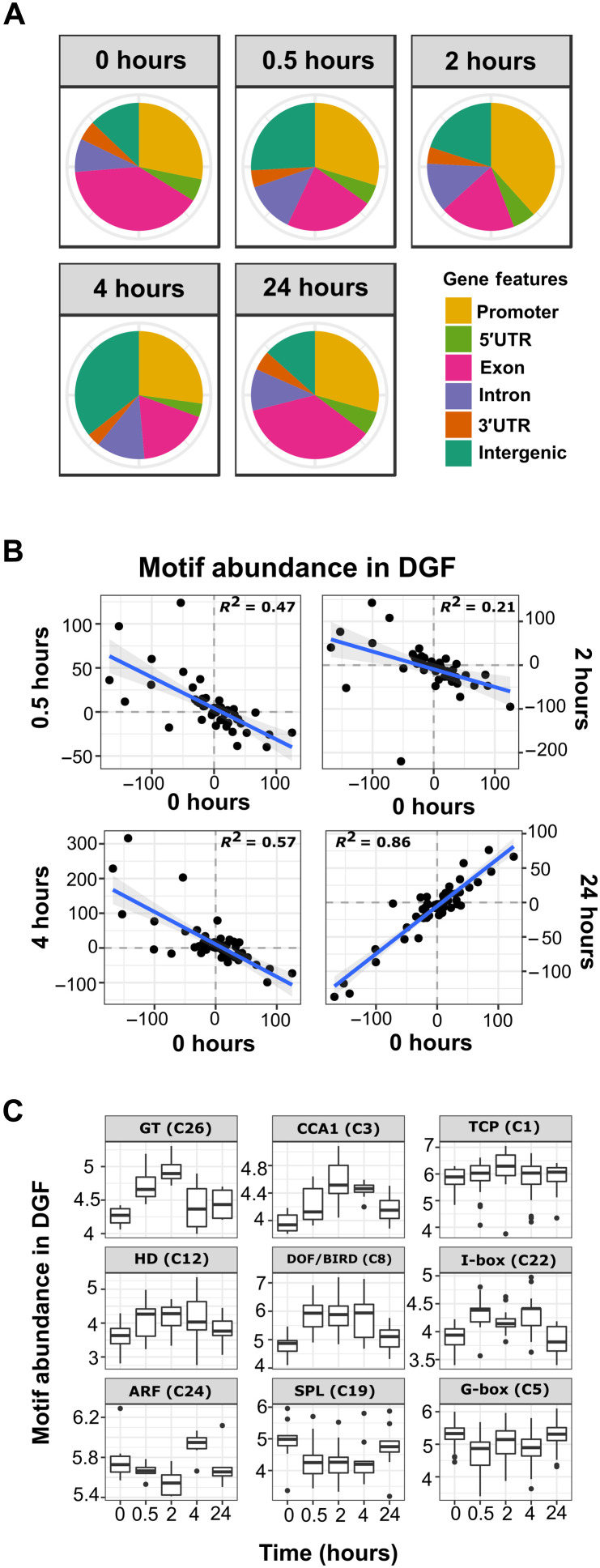
Transcription factor binding atlas for de-etiolating seedlings of *G. gynandra*. (**A**) Proportion of DGFs found in gene features at each time point. (**B**) Relationships between light samples and the dark sample for the relative bound motif abundances in DGFs. Negative correlations indicate that binding sites that are more abundant in the dark become less abundant in the light and vice versa. Values are the mean centered values for motif abundance across all five time points such that values greater than 0 indicate greater than average abundance at that particular time point. (**C**) Enrichment of motifs found within DGF regions associated with the most strongly induced genes between dark and 24 hours de-etiolation (pairwise *t* tests for each time point of motif groups are shown in table S6).

The proportion of DGF associated with gene features such as promoters, untranslated regions (UTRs), and exons changed during de-etiolation (Fig. 4A). For example, after 2 hours of light, a lower proportion of DGFs were found in exons but more were detected in promoters and introns compared with the dark (Fig. 4A). Only a small proportion of all motifs in open chromatin (DHS) were associated with DGF, but the extent to which the 49 groups of motifs were bound by transcription factors varied (fig. S4). For example, motifs associated with AP2/ERF binding showed the highest levels of occupancy, while AT-HOOK MOTIF CONTAINING NUCLEAR LOCALIZED (AHL) and NAC (NAM, ATAF and CUC) motifs showed almost no binding. Differences in the proportion of sites that were detected could be caused by variation in affinity or dependence on other transcription factors for DNA binding.

During de-etiolation, the extent of motif binding changed markedly. For example, after 0.5, 2, and 4 hours of light, motifs that tended to be bound in the dark were less likely to be bound (Fig. 4B). In contrast, those motifs less likely to be bound in the dark were more likely to be bound in the light (Fig. 4B). These relationships were reversed by 24 hours such that similar motifs were strongly bound at both 0 and 24 hours (Fig. 4B). These data argue for a dynamic response in transcription factor binding during the first few hours of light, with the similarity between binding at 0 and 24 hours, perhaps indicating control by the circadian clock. Separating these data according to the 49 groups of motifs associated with particular transcription factor families indicated that binding by GT-box, CIRCADIAN CLOCK ASSOCIATED 1 (CCA1), and TCP transcription factors peaked at 2 hours (Fig. 4C). Binding to HD, DNA-binding with one finger (DOF), BIRD/IDD, and I-box motifs showed a similar increase, but the peak at 2 hours was less clear (Fig. 4C). In contrast, motifs bound by Auxin response factors (ARF) transcription factors peaked at 4 hours, SPL motifs were most strongly bound in the dark and after 24 hours, and G-boxes showed a small decrease in binding at 0.5 hours compared with the dark.

From the data on chromatin accessibility (Fig. 3) combined with that relating to transcription factor binding (Fig. 4), we conclude the following. First, although increased accessibility can lead to greater numbers of motifs being available for binding, we did not always detect this binding taking place. This was the case for motif groups associated with BPC and WRKY transcription factor families. Second, and exemplified by TCP/HD and I-box motifs, in some cases, the greater number of motifs available for binding caused by broadening of DHS was also reflected in greater numbers of DGF in these regions. Last, while the number of motifs such as those associated with GT and CCA1 transcription factor families did not increase as a result of increased chromatin accessibility, the extent to which they were bound did increase (table S6). Overall, the data are consistent with de-etiolation affecting gene expression in *G. gynandra* through a complex interplay between changes in chromatin accessibility and transcription factor binding activities.

### C_4_ genes in *G. gynandra* are more strongly induced than orthologs in C_3_ *A. thaliana*

**Fig. 5.**
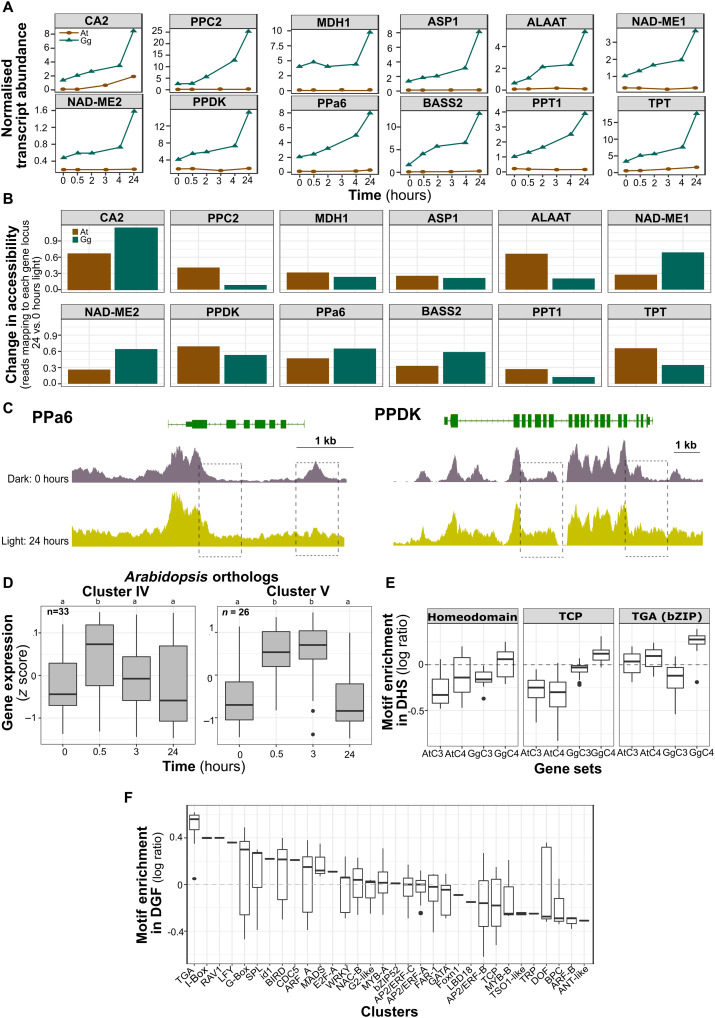
Comparative analysis of potential regulatory mechanisms for de-etiolating seedlings of C_3_ *A. thaliana* and C_4_ *G. gynandra*. (**A**) Changes in normalized transcript abundance of C_4_ genes from C_3_ *A. thaliana* and C_4_ *G. gynandra*. Each gene is depicted relative to the mean of all transcripts detected at each time point. (**B**) Changes in chromatin accessibility for C_4_ genes from C_3_ *A. thaliana* and C_4_ *G. gynandra* on light induction. Changes in accessibility were assessed by plotting the reads mapping to each gene locus at 24 versus 0 hours of light. (**C**) Schematic of *PPa6* and *PPDK* gene loci showing the dark and 24-hour light DNase I–seq chromatin accessibility traces. Read depth values within each box region were compared between the 24- versus 0-hour light. Regions within boxes were significantly more accessible in 24- versus 0-hour light (*P* values = 2.2 × 10^−16^) except for 3′ region of *PPa6*. (**D**) Distribution of expression levels of *A. thaliana* orthologs of highly and early induced transcription factors. Scaled expression values (*z* score) taken from Sullivan *et al.* (*21*) who sampled at 0, 0.5, 3, and 24 hours. (**E**) Motif groups that showed specific enrichment in the *G. gynandra* C_4_ cistrome. (**F**) Motif enrichment of DGFs from genes that showed the greatest induction during the 24-hour time course. Included in these 582 genes were most of the core C_4_ genes.

To better understand how these genome-wide patterns in chromatin accessibility and transcription factor binding may have affected on evolution of the C_4_ pathway, we compared our findings above with publicly available data from the C_3_ model *A. thaliana*. Reprocessing an analogous de-etiolation time course from *A. thaliana* (fig. S5) (*21*) and comparison with our *G. gynandra* data (Fig. 2) showed that, before being transfer to light, transcripts derived from C_4_ genes of *G. gynandra* were more abundant than orthologs from *A. thaliana* (Fig. 5A). Moreover, almost all C_4_ genes were induced more strongly in *G. gynandra* compared with *A. thaliana* (Fig. 5A). These data show that, in *G. gynandra*, the stronger expression of C_4_ genes is at least partly associated with a stronger response to light during de-etiolation. For some C_4_ genes [carbonic anhydrase (*CA2*), nicotinamide adenine dinucleotide (NAD)–dependent malic enzyme (*NAD-ME1* and *NAD-ME2*), pyrophosphorylase 6 (*PPa6*), and bile acid:sodium symporter family protein 2 (*BASS2*)], the stronger accumulation of C_4_ transcripts in C_4_ *G. gynandra* compared with C_3_ *A. thaliana* was associated with increased chromatin accessibility (Fig. 5B), but this was not always true. Some genes demonstrated complex changes in accessibility. In the case of the *PPa6* gene from *G. gynandra*, although an accessible region 3′ to the gene was lost, downstream of the first exon the gene body showed increased accessibility (Fig. 5C and table S6). In the case of pyruvate orthophosphate, dikinase (*PPDK*), a complex pattern emerged with much of the gene body being more accessible and some peaks becoming more defined by 24 hours of light (Fig. 5C and table S6).

As broad-scale chromatin patterns did not appear to clearly explain the strong induction of C_4_ genes, we next investigated trans-factors and cis-elements. We first wanted to understand the extent to which expression of trans-factors relevant to the light response differed between C_3_ *A. thaliana* and C_4_ *G. gynandra*. To do this, we compared the behavior of transcription factors that showed an early and robust induction to light (clusters IV and V in Fig. 2C) with orthologs in *A. thaliana*. Orthologs from *A. thaliana* to those in cluster IV (table S7) showed a clear peak at 0.5 hours that was not sustained (Fig. 5D), while those from cluster V (table S7) were induced for at least 3 hours (Fig. 5D) similar to that in *G. gynandra* (Fig. 2C). When *G. gynandra* orthologs for the most strongly induced transcription factors in *A. thaliana* at 0.5 versus 0 hours and at 3 versus 0 hours were identified, conservation in induction dynamics was also detected (fig. S6). These data are, therefore, consistent with the notion that the expression dynamics of transcription factors in networks associated with de-etiolation are conserved between C_3_ *A. thaliana* and C_4_ *G. gynandra*.

As the trans-factor network appears to have remained relatively stable since the last common ancestor of C_3_ *A. thaliana* and C_4_ *G. gynandra*, it seems possible that strong induction of C_4_ genes in the C_4_ leaf is associated with mutations in cis. To investigate this, we compared the composition of the cistrome of photosynthesis and C_4_ genes from *A. thaliana* and *G. gynandra*. Comparing the frequency of motifs in DHS associated with photosynthesis genes did not identify a specific motif or set of motifs that were absent in C_4_ genes from *A. thaliana* but present in C_4_ genes from *G. gynandra* as well as C_3_ photosynthesis genes from both species (fig. S7). This finding argues against a simple acquisition by C_4_ genes in *G. gynandra* of the cis-code that generates high levels of transcripts encoding the photosynthetic apparatus in the C_3_ leaf. However, it was notable that HD, TCP, and TGA (bZIP) motifs were enriched in the *G. gynandra* C_4_ cistrome (Fig. 5E). As C_4_ pathway genes need to be expressed either in mesophyll or bundle sheath cells, these motifs represent strong candidate regulators of the cell-preferential gene expression in C_4_ photosynthesis. While the total number of motifs present in DHS associated with C_4_ and C_3_ photosynthesis genes is sufficiently large to undertake statistical analysis for enrichment, far fewer DGFs were detected, and so this approach was not feasible. However, most of the C_4_ genes were found in the highly expressing module containing 582 genes that showed a strong expression at 24 hours of light (table S8), and the number of DGF in this module allowed statistical analysis. TGA motifs were most strongly enriched (Fig. 5F), suggesting that they may be linked to strong induction of C_4_ genes during de-etiolation. Other motifs enriched in the DGF of these strongly induced genes included the I-box and some, but not all, G-boxes (Fig. 5F). Overall, the data imply that the strong induction of C_4_ pathway genes during de-etiolation of *G. gynandra* can be associated with several factors. These include increased chromatin accessibility, acquisition of LREs in the cistrome, and increased binding of motifs associated with the response to light.

### LREs and strong induction of C_4_ genes in *G*. *gynandra*

**Fig. 6.**
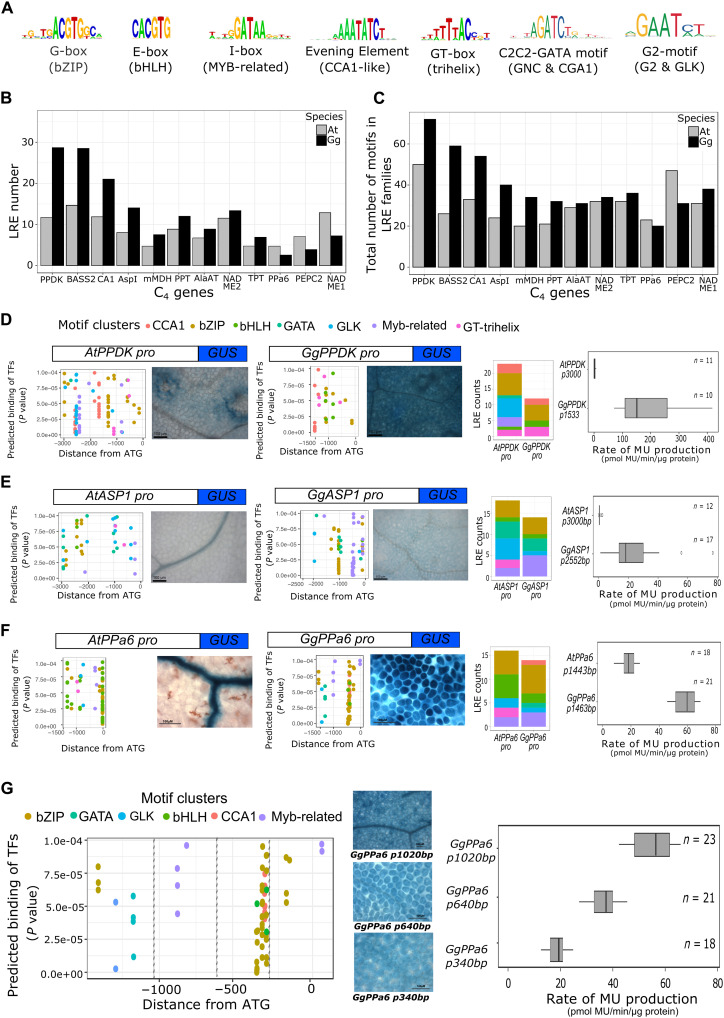
LREs are associated with strong induction of C_4_ genes. (**A**) Consensus motif sequence logo for seven transcription factor families associated with binding groups of LREs. (**B** and **C**) Bar plots representing the counts of individual LREs (B) and the counts of motifs from LRE families (C). Numbers plotted for 12 C_4_ genes in *A. thaliana* and *G. gynandra* from 1.5 kb upstream of the gene to the end of the coding sequence and nonoverlapping motif regions were counted (**D** to **F**). Scatter plots show position and predicted binding affinity of LRE motifs in 5′ region cloned for analysis, bar plots representing counts of LREs in regions cloned for analysis, representative images of stable transformants of *A. thaliana*, promoter::GUS fusions, and quantification of GUS activity via fluorometric MUG assay from *A. thaliana* and *G. gynandra* 5′ regions for *PPDK* (D), *ASPl* (E), and *PPa6* (F). For scatter plots (D to **G**), the *y* axis shows *P* values of matches between 5′ region and motif position weight matrices (PWMs), and the *x* axis shows position of the motif center relative to the translational start site. *P* values calculated by log-likelihood score using FIMO (*61*). Bar plots (D to G) show counts of nonoverlapping motifs colored by motif cluster. GUS images (D to G) are representative images of >10 independent T1 *Arabidopsis* lines. Scale bars represent 100 μm, and MUG assays are shown for multiple independent T1 *Arabidopsis* transformants with number of lines for each construct indicated.

From the RNA-seq and DNase I–seq analysis above, we therefore hypothesized that C_4_ genes in *G. gynandra* are strongly induced during de-etiolation because evolution has reinforced regulation by LREs. These sequences have previously been defined as G-, GT-, E-, and I-boxes and known to be bound by transcription factors in the bZIP, GT-trihelix, bHLH, and MYB-related families. We also included in this analysis master regulators of chloroplast biogenesis such as CCA1-like, GLK, GNC, and CGA1 (Fig. 6A). The genomic sequence of 9 of the 12 C_4_ genes from *G. gynandra* contained more LREs compared with orthologs in *A. thaliana* (Fig. 6B). As transcription factors bind variations to each basic motif (*26*), we used motif clustering to include sequences in our analysis that were closely related to each LRE. This provided a less biased analysis of potential binding sites in each promoter and also showed that most C_4_ genes from *G. gynandra* had more LREs than orthologs from *A. thaliana* (Fig. 6C). Good examples of this included the *PPDK* and aspartate aminotransferase (*ASP1*) genes from *G. gynandra*. To test whether promoter regions containing these LREs enable strong expression, we fused them to the reporter β-glucuronidase (GUS) and generated stable transformants of *A. thaliana*. In the case of *PPDK*, the promoter was shorter in *G. gynandra* because the upstream gene was closer (Fig. 6D), and so there were fewer LRE motifs. It was notable that members of MYB-related, G2-like, and GATA families were missing, while there were more motifs for GT-trihelix and bHLH families (Fig. 6D). Stable lines containing the *PPDK* promoter from *G. gynandra* showed a much stronger accumulation of GUS, and quantification of methyl umbelliferone (MU) confirmed this (Fig. 6D and fig. S8). It is, of course, possible that motifs not yet implicated in the response to light are present in the *G. gynandra PPDK* promoter, but we consider the GT-trihelix and bHLH as good candidates for allowing increased expression of *PPDK* in *G. gynandra* compared with *A. thaliana*. The *ASP1* promoter from *G. gynandra* contained fewer GT-trihelix and G2-like but more MYB-related motifs and was also much stronger than its ortholog from *A. thaliana* (Fig. 6E and fig. S9). Again, this sequence could contain previously unidentified motifs that are able to trigger a response to light. However, when combined with data from *PPDK*, these data suggest that evolution may have repurposed different LREs to up-regulate different C_4_ genes.

We also tested the promoter of *PPa6* where we did not detect an increase in the total number of LREs in the gene from *G. gynandra* compared with *A. thaliana* (Fig. 6E). In this case the *G. gynandra* promoter contained fewer GT-helix, G2-like, and GATA but more MYB-related, bZIP, and CCA1 motifs. The *PPa6* promoter from *G. gynandra* led to a substantially higher expression than that of *A. thaliana*, and this was associated with a domain shift from vascular cells to more ubiquitous expression in the leaf (Fig. 6F and fig. S10). As the *PPa6* promoters from *A. thaliana* and *G. gynandra* were of the same length, we created truncations of the latter to better understand the region responsible for increased expression. This indicated that motifs associated with bZIP, G2-like, and GATA families located between −1500 and −1000 base pairs (bp) upstream of the predicted translation start site were not necessary for increased expression (Fig. 6G). However, sequence between −950 and −640 bp containing motifs associated with members of the MYB-related family was required for increased expression, as was a region containing a cluster of CCA1, bZIP, and bHLH motifs between −400 and −340 bp upstream of the translation start site (Fig. 6G). The deletion of these motifs progressively reduced accumulation of GUS to levels similar to that derived from the *PPa6* promoter from *A. thaliana* (Fig. 6G). These data indicate that a sequence containing an I-box positioned −994 to −979 bp upstream of the predicted translation start as well as a group of bZIP, bHLH, and CCA1 motifs between −449 and −375 bp upstream of the predicted translation start is necessary for a strong expression from the *G. gynandra PPa6* promoter (Fig. 6G). It is also notable that none of these deletions affected the domain shift associated with expression of the *G. gynandra* promoter, and so we conclude that this is due elements in 340 bp upstream of the translation start site. Overall, the data from *PPDK*, *ASP1*, and *PPa6* indicate that a variety of mechanisms underpin a strong expression of C_4_ genes in *G. gynandra*. An increase in the total number of LREs appears not to be strongly correlated with increased C_4_ gene expression. However, the acquisition of specific and different LREs by each gene could have allowed higher expression. Moreover, and as exemplified by *PPa6*, regions containing LREs are necessary for a strong expression in mesophyll cells. Conducting these promoter reporter assays in *A. thaliana* shows that changes in cis to C_4_ genes are recognized by transcriptional networks that operate in the C_3_ plant. This is consistent with the notion that the altered expression of C_4_ genes is dependent on preexisting transcriptional networks.

## DISCUSSION

### Coordinated patterns of gene expression during de-etiolation of *G. gynandra*

The dark-to-light transition has been frequently used to understand the assembly of the photosynthetic apparatus (*27*). As cotyledons of *G. gynandra* operate C_4_ photosynthesis (*28*), de-etiolation of this species appeared as an attractive system with which to probe how C_4_ genes become coregulated with other photosynthesis genes. As expected from analysis of species using the ancestral C_3_ pathway (*20*, *29*–*31*), chloroplasts from *G. gynandra* followed a trajectory toward full photosynthetic competence in 24 hours of exposure to light. The induction of photosynthesis transcripts in *G. gynandra* following exposure to light was preceded by up-regulation of homologs of master regulators of de-etiolation including those involved in light and chloroplast regulation. This included the photomorphogenesis master regulator HY5 as well as the chloroplast regulators GNC and GLK2 (*32*). HY5 acts at the top of the transcriptional hierarchy and directly binds thousands of targets in *A. thaliana* (*33*) to integrate a number of inputs including light through its interaction with CONSTITUTIVE PHOTOMORPHOGENIC 1 (COP1) and subsequent degradation (*34*) as well as hormone pathways, leading to inhibition of auxin signaling to suppress hypocotyl elongation (*35*) and repression of ethylene signaling through the activation of the repressor ERF11 (*36*). Also consistent with analysis of C_3_ species (*37*) is the fact that transcripts derived from genes encoding negative regulators of de-etiolation such as phytochrome interacting factor 7 (PIF7), PIF3-like 5, and other bHLH family members from *G. gynandra* became less abundant after exposure to light. Comparison with a publicly available time course from *A. thaliana* (*21*) indicated similar changes to abundance of transcripts encoding transcription factors in the first hour of light exposure, therefore strongly suggesting that a major remodeling of these regulators in trans is not associated with C_4_ evolution.

### Chromatin dynamics and transcription factor binding during de-etiolation of *G. gynandra*

DNase I–seq, MNase (micrococcal nuclease digestion) sequencing, and ATAC (transposase-accessible chromatin) sequencing allow regions of open chromatin to be defined (*38*). In plants, such technologies have been used to better understand gene expression associated with processes including de-etiolation and heat stress (*21*), the cold response (*39*), and how chromatin accessibility affects gene expression across *A. thaliana* ecotypes (*40*). Defining regions of open chromatin allows motifs available to be bound by transcription factors to be predicted in silico (*21*). Previous analysis of leaves from C_3_ and C_4_ plants identified gene expression patterns and DHS in mature leaves in which photosynthesis had already been established (*41*, *42*). Here, we used de-etiolation to provide insight into how genome-wide patterns of chromatin changed during dark-to-light transition in seedling of C_4_ plant *G. gynandra*. Genome-wide chromatin accessibility increased most strongly immediately upstream of the predicted TSSs consistent with previous analysis of mature leaves of *A. thaliana* after transfer from dark to light (*43*).

In addition to defining regions of chromatin that could be bound by transcription factors, we also undertook deep sequencing to generate in vivo evidence of protein-DNA interactions. It has been proposed that this approach can generate base pair resolution information on transcription factor binding sites (*44*), but leaves contain many cell types differing in gene expression, likely leading to DGF representing estimates of binding. Despite this, we identified 263,404 DGFs in *G. gynandra* during de-etiolation that compares favorably with 282,030 DGFs in a publicly available dataset for de-etiolation of *A. thaliana* (*21*). As would be expected, the increased accessibility of chromatin immediately upstream of genes after transfer from dark to light was reflected in an increased number of DGF being detected in promoter sequence. Moreover, while fewer DGFs were detected in exons after transfer to light, those in introns increased. In *Drosophila melanogaster* enhancers have been reported to be depleted in exons but enriched in promoters, 5′UTRs, and introns (*45*). Transcription factor binding sites in coding sequence have been termed duons as they determine both gene expression and the amino acid code, and it has been proposed that they act as repressors of gene expression in humans (*46*). It is therefore possible that, in plants, exons are biased toward harboring motifs that repress gene expression. Consistent with this, duons placed downstream of the constitutive *CaMV35S* promoter repressed expression in mesophyll cells (*9*, *10*). The extent to which duons act as repressors of gene expression in plants remains to be determined.

### C_4_ transcripts of *G. gynandra* are induced rapidly and strongly during de-etiolation

The principle that, in a C_4_ leaf, genes encoding proteins of the C_4_ pathway must become subject to gene regulatory networks allowing co-regulation with C_3_ photosynthesis genes is intuitive, but, to our knowledge, strong evidence for this has been lacking. Our data clearly indicate that, after *G. gynandra* is exposed to light, accumulation of transcripts encoding photosynthesis proteins and components of the C_4_ cycle is strong and coordinated. In contrast, reanalysis of publicly available data (*21*) showed that accumulation of transcripts derived from orthologs to C_4_ genes in *A. thaliana* was much lower. Our findings from these dicotyledonous species are consistent with reanalysis of publicly available data (*47*) from C_4_ maize and C_3_ rice (fig. S11). We conclude that, in two lineages that have independently evolved C_4_ photosynthesis, genes associated with the C_4_ cycle become part of gene regulatory networks that respond very strongly to the dark-to-light transition associated with de-etiolation. The most parsimonious explanation for these findings is that, in C_3_ plants, genes encoding components of the C_4_ pathway show a basal induction in response to light during de-etiolation and that, during the evolution of C_4_ photosynthesis, this ancestral system becomes amplified.

To better understand changes that may have led to such a gain in amplitude, we assessed the cistrome of C_4_ pathway genes and compared this to those of C_3_ photosynthesis genes in both *G. gynandra* and *A. thaliana* as well as C_4_ orthologs in *A. thaliana*. This indicated that LREs associated with photosynthesis genes in C_3_ *A. thaliana* have been acquired by C_4_ pathway genes of *G. gynandra* and may contribute to increased accumulation of their transcripts compared with C_4_ orthologs in *A. thaliana*. For example, G-box motifs were enriched in the cistrome of C_4_ pathway genes from *G. gynandra* as well as that of C_3_ photosynthesis genes from *A. thaliana*. Among the G-box motifs, the most enriched was that associated with HY5 binding; however, this motif is, to varying degrees, enriched in all four of the cistromes, suggesting that it does not have a special role in C_4_ photosynthesis. In addition to a gain of LREs in C_4_ cycle genes, motifs associated with TGA, HD, and TCP transcription factor families were enriched in the cistrome of C_4_ cycle genes. The DNase I assay also provided evidence for binding by these transcription factor families. To our knowledge, these transcription factors have not previously been linked to de-etiolation of photosynthesis. Rather, the TGA family of transcription factors has been linked to plant defense (*48*) and has been involved in boundary patterning in leaf development (*49*), and HD factors can be involved in, for example, vascular development (*50*) and anthocyanin production (*51*). It, therefore, seems unlikely that enrichment of these motifs is related to enhanced accumulation of C_4_ cycle transcripts in the C_4_ leaf. However, some members of these families are preferentially expressed in either mesophyll or bundle sheath cells of leaves of *G. gynandra* (fig. S12), and so they may well be important for patterning C_4_ gene expression to either mesophyll or bundle sheath cells.

In summary, our analysis of molecular events taking place during de-etiolation of *G. gynandra* provides the following insights into the evolution of C_4_ photosynthesis. First, the data are consistent with multiple mechanisms, allowing the enhanced accumulation of C_4_ transcripts in C_4_ compared with C_3_ leaves. We found no evidence for large-scale remodeling of transcription factors associated with the induction of photosynthesis genes in response to light. However, for C_4_ genes such as those encoding CA2, NAD-ME1 and NAD-ME2, PPa6, and BASS2, chromatin accessibility increased. As the C_4_ proteins phosphoenolpyruvate carboxykinase (PEPCK) and PPDK function during early seedling growth to allow remobilization of carbon skeletons for gluconeogenesis and the genes encoding them are strongly expressed (*52*), it is perhaps expected that increased chromatin accessibility was not detected for all C_4_ cycle genes. Alongside alterations to chromatin accessibility for some C_4_ genes, we also found a gain in the LREs associated with the response of gene expression to light. However, rather than an increase in the total number of LREs in C_4_ genes from *G. gynandra* compared with *A. thaliana* being functionally important, our data point toward specific families of LREs being relevant to different genes. For example, although the promoters of *PPDK* and *ASP1* from *G. gynandra* contained distinct LREs (E- and GT-boxes, or I-boxes, respectively), they both allowed high expression. For the *PPa6* gene from *G. gynandra*, promoter deletion analysis showed that regions containing G- and I-boxes were necessary for a strong expression. Overall, the data support a model in which evolution has made use of distinct LREs to up-regulate different C_4_ genes in *G. gynandra*. We conclude that, during the evolution of C_4_ photosynthesis, changes to the cis-code, including the acquisition of LREs by C_4_ genes, facilitated the rewiring of gene expression and allowed their integration into ancestral transcriptional networks associated with the induction of photosynthesis in C_3_ plants. Most previous work on understanding the transcriptional regulation of C_4_ photosynthesis (*7*–*16*) has focused on mechanisms, allowing the cell-specific expression of genes in C_4_ leaves compared with the ancestral C_3_ state. To our knowledge, there were no data available from closely related C_3_ and C_4_ species on the molecular rewiring of gene induction and chromatin accessibility. Thus, the present work provides initial insights into this key process through an integrated analysis of changes in transcript abundance, chromatin accessibility, and in vivo transcription factor binding. Combined with functional testing through promoter deletions, these data support the conclusion that the evolution of strong expression of C_4_ photosynthesis genes is associated with the acquisition of LREs.

## MATERIALS AND METHODS

### Plant growth, chlorophyll quantitation, and microscopy

G*. gynandra* seeds were sown directly from intact pods and germinated on moist filter papers in the dark at 32°C for 24 hours. Germinated seeds were then transferred to half-strength Murashige and Skoog medium with 0.8% (w/v) agar (pH 5.8) and grown for 3 days in a growth chamber at 26°C. De-etiolation was induced by exposure to white light with a photon flux density of 350 μmol m^−2^ s ^−1^ and photoperiod of 16 hours. Whole seedlings were harvested at 0.5, 2, 4, and 24 hours after illumination (starting at 8:00 a.m. with light cycle 6:00 a.m. to 10:00 p.m.). Tissue was flash-frozen in liquid nitrogen and stored at −80°C before processing.

For analysis of chlorophyll content, de-etiolating *G. gynandra* seedlings were flash-frozen at 0, 0.5, 2, 4, or 24 hours after light exposure. Tissue (100 mg) was suspended in 1 ml of 80% (v/v) acetone at 4°C for 10 min before centrifugation at 15,700*g* for 5 min and removal of the supernatant. The pellet was resuspended in 1 ml of 80% (v/v) acetone at 4°C for 10 min, precipitated at 15,700*g* for 5 min. Supernatants were pooled, and absorbance measured in a spectrophotometer at 663.8 and 646.6 nm. Total chlorophyll content determined as described previously (*53*). For electron microscopy, *G. gynandra* cotyledons (~2 mm^2^) were excised with a razor blade and fixed immediately in 2% (v/v) glutaraldehyde and 2% (w/v) formaldehyde in 0.05 to 0.1 M sodium cacodylate (NaCac) buffer (pH 7.4) containing 2 mM calcium chloride. Samples were vacuum-infiltrated overnight, washed five times in deionized water, and postfixed in 1% (v/v) aqueous osmium tetroxide and 1.5% (w/v) potassium ferricyanide in 0.05 M NaCac buffer for 3 days at 4°C. After osmication, samples were washed five times in deionized water and postfixed in 0.1% (w/v) thiocarbohydrazide in 0.05 M NaCac buffer for 20 min at room temperature in the dark. Samples were then washed five times in deionized water and osmicated for a second time for 1 hour in 2% (v/v) aqueous osmium tetroxide in 0.05 M NaCac buffer at room temperature. Samples were washed five times in deionized water and subsequently stained in 2% (w/v) uranyl acetate in 0.05 M maleate buffer (pH 5.5) for 3 days at 4°C and washed five times afterward in deionized water. Next, samples were dehydrated in an ethanol series and transferred to acetone and then to acetonitrile. Samples were embedded in a Quetol 651 resin mix (TAAB Laboratories Equipment Ltd.). For transmission electron microscopy, ultrathin sections were cut with a diamond knife, collected on copper grids, and examined in a FEI Tecnai G2 transmission electron microscope (200 keV, 20-μm objective aperture). Images were obtained with Advanced Microscopy Techniques (AMT) charge-coupled device (CCD) camera. For scanning electron microscopy (SEM), ultrathin sections were placed on plastic coverslips that were mounted on aluminum SEM stubs, sputter-coated with a thin layer of iridium, and imaged in a FEI Verios 460 scanning electron microscope. For light microscopy, thin sections were stained with methylene blue and imaged by an Olympus BX41 light microscope with a mounted MicroPublisher 3.3 Real Time Viewing (RTV) camera (QImaging).

### RNA-seq and DNase I–seq

Before processing, frozen samples were divided into two: the first being used for RNA-seq analysis and the second for DNase I–seq. Samples were ground in a mortar and pestle, and RNA extraction was carried out with the RNeasy Plant Mini Kit (74904, QIAGEN) according to the manufacturer’s instructions. RNA quality and integrity were assessed on a Bioanalyzer High Sensitivity DNA Chip (Agilent Technologies). Library preparation was performed with 500 ng of high integrity total RNA (RNA integrity number > 8) using the QuantSeq 3′ mRNA-Seq Library Preparation Kit FWD for Illumina (Lexogen) following the manufacturer’s instructions. Library quantity and quality were checked using Qubit (Life Technologies) and a Bioanalyzer High Sensitivity DNA Chip (Agilent Technologies). Libraries were sequenced on NextSeq 500 (Illumina, Chesterford, UK) using single-end sequencing and a Mid Output 150 cycle run.

To extract nuclei, tissue was ground in liquid nitrogen and incubated for 5 min in 15 mM Pipes (pH 6.5), 0.3 M sucrose, 1% (v/v) Triton X-100, 20 mM NaCl, 80 mM KCl, 0.1 mM EDTA, 0.25 mM spermidine, 0.25 g of polyvinylpyrrolidone (Sigma-Aldrich), and EDTA-free proteinase inhibitors (Roche); filtered through two layers of Miracloth (Millipore); and pelleted by centrifugation at 4°C for 15 min at 3600g. To isolate deproteinated DNA, 100 mg of tissue from seedlings exposed to 24-hour light was harvested 2 hours into the light cycle, 4 days after germination. DNA was extracted using the QIAGEN DNeasy Plant Mini Kit (QIAGEN, UK) according to the manufacturer’s instructions. Nuclei (2 × 10^8^) were resuspended at 4°C in digestion buffer [15 mM tris-HCl, 90 mM NaCl, 60 mM KCl, 6 mM CaCl_2_, 0.5 mM spermidine, 1 mM EDTA, and 0.5 mM EGTA (pH 8.0)]. DNase I (Fermentas) at 2.5 U was added to each tube and incubated at 37°C for 3 min. Digestion was arrested by adding a 1:1 volume of stop buffer [50 mM tris-HCl, 100 mM NaCl, 0.1% (w/v) SDS, 100 mM EDTA (pH 8.0), 1 mM spermidine, 0.3 mM spermine, and ribonuclease A (40 μg/ml)] and incubated at 55°C for 15 min. Proteinase K (50 U) was then added, and samples were incubated at 55°C for 1 hour. DNA was isolated by mixing with 1 ml of 25:24:1 phenol:chloroform:isoamyl alcohol (Ambion) and spun for 5 min at 15,700*g* followed by ethanol precipitation of the aqueous phase. Samples were size-selected (50 to 400 bp) using agarose gel electrophoresis and quantified fluorometrically using a Qubit 3.0 Fluorometer (Life technologies), and a total of 10 ng of digested DNA (200 pg liter^−1^) was used for library construction. Sequencing ready libraries were prepared using a TruSeq Nano DNA library kit according to the manufacturer’s instructions. Quality of libraries was determined using a Bioanalyzer High Sensitivity DNA Chip (Agilent Technologies) and quantified by Qubit (Life Technologies) and quantitative polymerase chain reaction using the NGS Library Quantification Kit (KAPA Biosystems) before normalization and then pooled, diluted, and denatured for paired-end sequencing using High Output 150 cycle run (2 × 75 bp reads). Sequencing was performed using NextSeq 500 (Illumina, Chesterford, UK) with 2 × 75 cycles of sequencing.

### RNA-seq data processing and quantification

Commands used are available on Dryad and GitHub (“command_line_steps”), but an outline of steps was as follows. Raw single-end reads were trimmed using trimmomatic (version 0.36) (*54*). Trimmed reads were then quantified using salmon (version 0.4.234) (*55*) after building an index file for a modified *G. gynandra* transcriptome. The transcriptome was modified to create a pseudo-3′UTR sequence of 339 bp (the mean length of identified 3′UTRs) for *G. gynandra* gene models that lacked a 3′UTR sequence that was essentially an extension beyond the stop codon of the open reading frame. Inclusion of this psuedo-3′UTR improved mapping rates. Each sample was then quantified using the salmon “quant” tool. All *.sf files had the “NumReads” columns merged into a single file (All_read_counts.txt) to allow analysis with both DEseq2 (*56*) and edgeR (*57*). The edgeR pipeline was run as the edgeR.R R script (on Dryad and GitHub) on the All_read_counts.txt file to identify the significantly DEGs by comparing each time point to the previous one. A low-expression filter step was also used. We then similarly analyzed the data with the DEseq2 package using the DEseq2.R R script (on Dryad and GitHub) on the same All_read_counts.txt file. This also included the principal components analysis shown in Fig. 2A. The intersection from both methods was used to identify a robust set of differentially regulated genes. For most further analysis of the RNA-seq data, mean transcripts per million (TPM) values for each time point (from three biological replicates) was first quantile-normalized, and then, each value was divided by the sample mean such that a value was of 1 represented the average for that sample. This processing facilitated comparisons between experiments across species in identifying changes to transcript abundance between orthologs.

### Identification of C_3_ and C_4_ gene lists and heatmap plotting

To map orthologs between *A. thaliana* and *G. gynandra*, OrthoFinder (*58*) was used. This allows more complex relationships than a 1:1 to be identified and placed into orthogroups. C_3_ photosynthesis genes were first identified from *A. thaliana* through the “photosynthesis” (GO:0015979) keyword search on the TAIR browse tool (www.arabidopsis.org/servlets/TairObject?type=keyword&id=6756) and resulted in 92 genes for *A. thaliana*. Their orthologs were found in *G. gynandra* using the orthogroups generated between the two species and resulted in 93 C_3_ photosynthesis genes. C_4_ genes used in this study are considered the “core” pathway genes and are a manually curated set based on previous analysis (*17*). Initially, multiple paralogs were included but noninduced transcripts were then filtered out. Orthologs between the two species were again identified from the orthogroups from OrthoFinder. The *G. gynandra* C_3_ and C_4_ gene quantile-normalized expression values were further processed with each value being divided by the row mean and log_10_(x/row mean) plotted as a heatmap using the R script Fig2B.R on data file Fig2B_heatmap_data.txt (on Dryad and GitHub). The heatmap for *A. thaliana* C_3_ and C_4_ gene expression was made in the same way as for the *G. gynandra* data using the gene lists as previously described.

To generate the heatmap of all highly variable transcription factors, all putative transcription factors were extracted from the DEGs when comparing each time point to the previous one. All potential transcription factors in *G. gynandra* were obtained by blastp searching with all protein sequences from the Arabidopsis Plant Transcription Factor Database (http://planttfdb.gao-lab.org/download/TF_list/Ath_TF_list.txt.gz) with command available on Dryad and GitHub. All predicted transcription factors found in the DEGs had their normalized TPM values converted to *z* scores (by row) and plotted using the heatmap.2 R package using the Fig2C.R script with the Fig2C_ heatmap_data.txt file. Clusters were manually assigned from the hierarchical clustering and word clouds made of transcription factor family names for each cluster. *A. thaliana* orthologs (identified using OrthoFinder) had their expression patterns plotted in de-etiolation time course from Sullivan *et al.* (*21*).

### DNase I–seq data processing

Three biological replicates for each time point were sequenced in multiple runs with one sample being chosen, based on initial QC scores, for deeper sequencing to provide the necessary depth for calling both DHS and DGFs. For each sample, raw reads from multiple sequencing runs were combined and trimmed for low-quality reads using trimmomatic. These files were analyzed with fastqc (www.bioinformatics.babraham.ac.uk/projects/fastqc/) to ensure samples passed QC parameters (see Ggynandra_DNaseSEQ_multiqc_report.html on Dryad and GitHub) and then mapped to the *G. gynandra* genome using bowtie2 (version 2.3.4.1) with the “--local” preset option. Following mapping, a bash script (DNaseSEQ_tagAlign.sh on Dryad and GitHub) was run on each bam file that, in summary, filters low-quality (MAPQ <30) mapped reads and plots MAPQ distribution; removes duplicates; measures library complexity, fragment sizes, and GC bias; and lastly makes tagAlign files. The DHS from each of the three replicates per time point were compared to ensure that they showed consistency (overlapping) that showed one replicate from the 4 hour time point was an “outlier” showing little overlap with the other two replicates and was therefore removed from downstream analysis. The remaining replicates tagAlign files from each time point were then merged before running another bash script (DHS_DGF_identification.sh on Dryad and GitHub) for each time point that, in summary, uses “macs2 callpeak” to identify “narrowPeaks”, finds the distance for each DHS to its closest TSS, and calculates SPOT scores (see www.encodeproject.org/data-standards/dnase-seq/). Before calling DGF, we identified “hyperconserved” DHS as those that were present in each time point and any DHS that were light specific (being found in all four-light time points but not in the dark). Within these regions, DGFs were called using the wellington_footprints.py program (*59*). The footprints identified with a log(*P* value) cutoff of <−10 were used for further analysis.

To carry out comparative analysis between *G. gynandra* and *A. thaliana*, an analogous de-etiolation time course (*21*) was reprocessed in the same way as the *G. gynandra* data. *A. thaliana* DNase I–seq data were mapped to the TAIR9 genome, and RNA-seq was mapped using Salmon to the Araport11 transcriptome, followed by the use of tximport to collapse expression values for all isomers into a single value, a step not required for *G. gynandra* as it lacks isomer information. To allow interspecies comparisons, as with *G. gynandra* the *A. thaliana*, RNA-seq data were quantile-normalized, and then, each value divided by the samples mean expression value.

### Chromatin dynamics at gene loci and correlation of motif abundance between time points

Raw DNase-seq reads were analyzed around gene loci using 2000 bp up- and downstream of the TSS. First, a bed file was generated with regions centered on each TSS with 2000 bp in each direction. Then, each raw pooled (from three replicates) DNase-seq file was normalized using deepTools (bamCoverage --binSize 1 --normalizeUsing RPKM) (*60*), and, for time point comparisons, the log_2_ fold was calculated for each position from these normalized count values using deepTools “bamCompare” with the output files converted to bed format. An R script (on Dryad and GitHub) then calculated and plotted the mean values at each position across the 4000 bp TSS centered regions of each gene loci. This was using normalized values for the dark sample for Fig. 3B and using the log_2_ values generated for the light time point versus dark comparisons.

Motif frequencies were obtained for all DGF at each time point. These values were then normalized to account for differences in total DGF identified and allow comparisons between time points. Then, the mean frequency for each motif group was calculated per time point. These values were lastly mean centered across the five time points such that positive values represented higher than average levels and were plotted.

### Motif analysis of DHS regions and DNase I bias correction

To identify enriched motifs in the cistromes of the DEGs from the 0.5, 2, and 4 hour time points, we first scanned for the presence of motifs from the Non-Redundant Core Plant Motif Database (https://jaspar2018.genereg.net/downloads/) using the meme suite FIMO tool (*61*) in all hyperconserved DHS regions that intersected with low-expression–filtered gene loci (gene body or 1.5–kilo-bp promoter region). This formed a background of motif frequencies from which expected values could be calculated for enrichment analyzes. For each gene set analyzed, the frequency of motifs was compared against the expected values and the log ratio of these calculated such that positive values represented motifs found more frequently than expected. All motifs were analyzed in semiredundant motif groups based on motif clustering using the RSAT motif clustering tool (http://rsat.sb-roscoff.fr/matrix-clustering_form.cgi). These groups typically, but not always, represented motifs that are recognized by members of the same transcription factor (TF) family and, therefore, are likely to be bound by many members of these gene families.

To reduce the proportion of false-positive DGF calls caused by DNase I cutting bias, DNase I–seq was performed on deproteinated genomic DNA (gDNA) and mapped to the *G. gynandra* genome. The hexamer cutting frequencies at the DNase I cutting sites were used to generate a background signal profile that was incorporated into a mixture model to calculate the log- footprint likelihood ratio (FLR) for each footprint using the R package MixtureModel (*25*) DGFs with low confidence (FLR < 0) were filtered out resulting in a reduction of 11 to 37% of DGF per time point. Same pipeline was used in previous analysis (*42*). The pipeline is illustrated in fig. S3.

### DGF genomic feature distributions and DGF motif frequencies

To identify the distribution of DGF across genomic features, we used bedtools intersect to find the frequency of intersection between the DGF with features in the genome annotation gff3 file promoter (2000 bp upstream of TSS), 5′UTR, coding sequence (CDS), intron, 3′UTR, and intergenic). These frequencies were then plotted but also further processed to understand the density of DGF rather than simply frequency as some gene features (such as the 2000-bp promoters) were larger than others. To determine the densities, the frequencies were divided by the total length of each feature across the genome. Before motif searching in DGF, each DGF region was extended by 4 bp in each direction and scanned for motifs as described for the DHS regions. For each motif, the proportion was found for each time point to compensate for variations in sequencing depth and total number of DGF identified. These proportions from the four light samples were compared with the dark to highlight how global transcription factor binding patterns related to the dark and how this evolved following exposure to light. These abundances were then also compared across the time series by grouping the motifs into related motif groups, as described for motif analysis of DHS, showing how related transcription factor binding activity changed following exposure to light.

### Change in accessibility analysis and motif enrichment

Genomic regions were defined for each of the loci for C_4_ pathway genes in *G. gynandra* and their orthologs in *Arabidopsis*. Within these regions, the mean normalized read depth for each locus in each species was calculated for the dark and 24 hours after light exposure samples. Then, the fold change was calculated (24 hours in the dark) to quantify the overall increase in accessibility over the loci after the 24 hours. Comparing these values for orthologous genes in the two species indicates whether the genes become more accessible in response to light.

For various gene set analyses of motif enrichments, motifs that fell within the gene loci including the putative promoter area were assigned to these genes. When comparing across time points, their relative abundances between time points within these subsets were analyzed. When analyzing a gene set across all time points, the abundances of motifs were compared against the abundance in all DHS or DGF regions. This background allows an expected proportion to be calculated with “enrichment” defined as a higher proportion than expected from this background and “depletion” as a lower proportion than expected.

### Identification of conserved transcription factor responses following light exposure

Following gene expression clustering of *G. gynandra* transcription factors, the two clusters showing strong and rapid induction to light were identified. Taking these genes, their orthologs were found in *Arabidopsis* using OrthoFinder orthogroups. These *Arabidopsis* orthologs were then found in the *Arabidopsis* de-etiolation dataset (*21*), and their expression patterns were plotted showing relatively high conservation in rapid light responsiveness.

### Phylogeny and cell-specific expression of HD factors in *A. thaliana* and *G. gynandra*

HD factors were identified from *A. thaliana* transcription factor databases, and all potential transcription factors in *G. gynandra* were identified by sequence similarity. Phylogenetic trees of the protein sequences from both species were made using the ete3 tool. The tree was loaded into the iTOL web tool where the log(BS/M or whole leaf) ratio of each gene was added to the tree. This expression data were obtained from publicly available datasets (*62*, *63*).

### Reprocessing of *Oryza sativa* and *Z. mays* de-etiolation time course RNA-seq

Data from the monocot de-etiolation study (*47*) were downloaded from the Short Read Archive (SRX766219). Reads for both species were quantified using Salmon quant with the *Z. mays* reads being mapped to Zm-B73-REFERENCE-NAM-5.0_Zm00001e.1.cdna.fa file available from MaizeDB, while *O. sativa* reads were mapped to Osativa_323_v7.0.cds_primaryTranscriptOnly.fa available from Phytozome. TPM values were quantile-normalized, and then, each value divided by the sample mean. *O. sativa* C_4_ orthologs were identified using OrthoFinder to identify orthogroups with the *A. thaliana* C_4_ orthologs used in this study*. Z. mays* C_4_ genes were identified by blasting to these same *A. thaliana* genes. Line plots were then made grouping all putative orthologs.

### Cloning, GUS staining, and MUG assays

Following procedure previously described (*64*), promoter fragments for *A. thaliana* and *G. gynandra PPDK*, *ASP1*, and *PPa6* were amplified from *A. thaliana* Col-0 and *G. gynandra* gDNA and cloned into the pENTR entry vector using TOPO-D cloning as per manufacturer’s instructions (Invitrogen). To fuse promoter fragments with the *uidA* GUS reporter, a Gateway LR (Invitrogen) reaction was performed. Fragments were transferred from the pENTR vector into a modified pGWB3 vector (*65*) containing an intron in the *uidA* sequence. The promoter cut-down constructs for *G. gynandra PPa6* were made using the Golden Gate cloning system (*66*). The promoter sequences were cloned from *G. gynandra* gDNA, domesticated to remove Bpi I and Bsa I restriction sites, and cloned into level 0 vectors. Level 1 constructs were generated to express candidate promoter and a *uidA* GUS reporter. These level 1 constructs were assembled into level 2 modules with spectinomycin selection (*67*). All constructs were then transformed into *Agrobacterium tumefaciens* strain GV3101 by electroporation and introduced into *A. thaliana* Col-0 by floral dipping (*68*). To assess the degree of induction of *A. thaliana* and *G. gynandra* C_4_ genes, promoter sequence of *PPDK*, *ASPI*, and *PPa6* was used for uidA fusion. GUS staining was undertaken on at least 10 randomly selected T1 plants. The staining solution contained 0.1 M Na_2_HPO_4_ (pH 7.0), 2 mM potassium ferricyanide, 2 mM potassium ferrocyanide, 10 mM EDTA (pH 8.0), 0.06% (v/v) Triton X-100, and X-gluc (0.5 mg ml^−1^). Leaves from 3-week-old plants were vacuum-infiltrated three times in GUS solution for 1 min and then incubated at 37°C for 12 to 16 hours depending on the strength of the promoter being assessed. Next, stained samples were fixed in 3:1 (v/v) ethanol:acetic acid for 30 min at room temperature, cleared in 70% (v/v) ethanol at 37°C, and then placed in 5 M NaOH for 2 hours. The samples were stored in 70% (v/v) ethanol at 4°C. The samples were imaged with an Olympus BX41 light microscope with QCapture Pro 7 software and a QImaging MicroPublisher 3.3 RTV camera. To quantify reporter accumulation from each promoter, the quantitative assay that assesses the rate of 4-Methylumbelliferyl glucuronide (MUG) conversion to 4-methylumbelliferone (MU) was performed on between 10 and 25 lines. The tissue was frozen in liquid nitrogen and homogenized, and soluble protein was extracted in five volumes of protein extraction buffer [1 mM MgCl_2_, 100 mM NaCl, and 50 mM tris (Melford) (pH 7.8)]. Then, 15 μl of protein extract was incubated with 60 μl of MUG at 37°C for 1, 2, 3, and 4 hours. The reaction was stopped after each time point by the addition of 75 μl of 200 mM anhydrous sodium carbonate.

GUS activity was analyzed via measurements of fluorescence of MU at 455 nm after excitation at 365 nm. The concentration of MU/unit fluorescence in each sample was interpolated using a concentration gradient of MU over a linear range.

## Notes

View/request a protocol for this paper from *Bio-protocol*.
