# Supplementary material for: C4 gene induction during de-etiolation evolved through changes in cis to allow integration with ancestral C3 gene regulatory networks
Source: Sci Adv. 2023 Mar 29;9(13):eade9756. doi: 10.1126/sciadv.ade9756 (PMC10058240; doi:10.1126/sciadv.ade9756)
Supplement: Supplementary file 1 — Figs. S1 to S12 Legends for tables S1 to S8 [file sciadv.ade9756_sm.pdf]

Science Advances
AAAS

Supplementary Materials for

C_4 gene induction during de-etiolation evolved through changes in cis to allow integration with ancestral C_3 gene regulatory networks

Pallavi Singh *et al.*

Corresponding author: Pallavi Singh, ps753@cam.ac.uk; Julian M. Hibberd, jmh65@cam.ac.uk

Sci. Adv. **9**, eade9756 (2023)
DOI: 10.1126/sciadv.ade9756

The PDF file includes:

Figs. S1 to S12
Legends for tables S1 to S8

Other Supplementary Material for this manuscript includes the following:

Tables S1 to S8

fig. S1

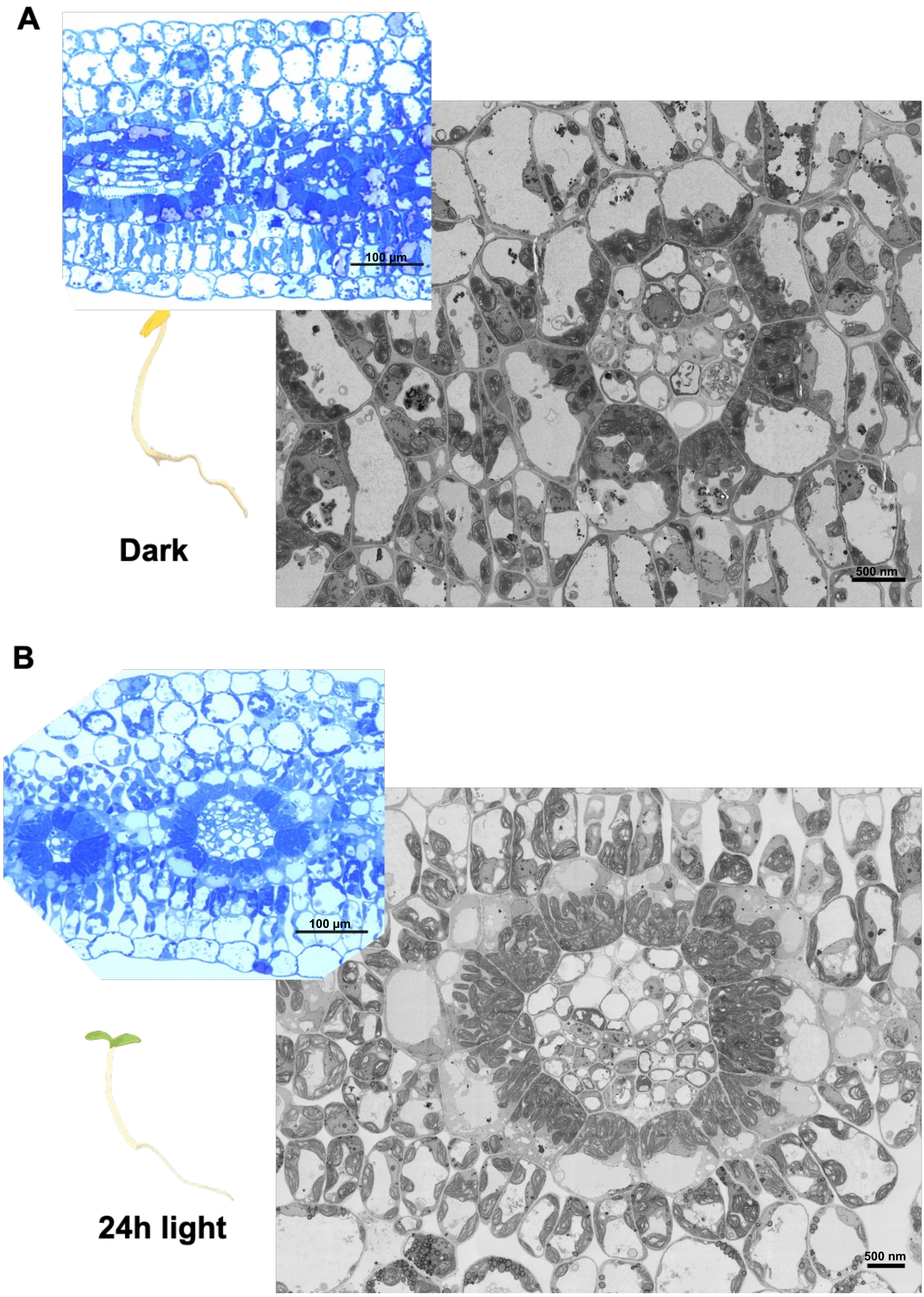

fig. S1: Representative bright field and scanning electron microscope (SEM) images from *G. gynandra* cotyledons at 0 hours (a) and 24 hours (b) after exposure to light. Scale bars represent 100 µm for light microscope images, and 500 nm for SEM images.

fig. S2

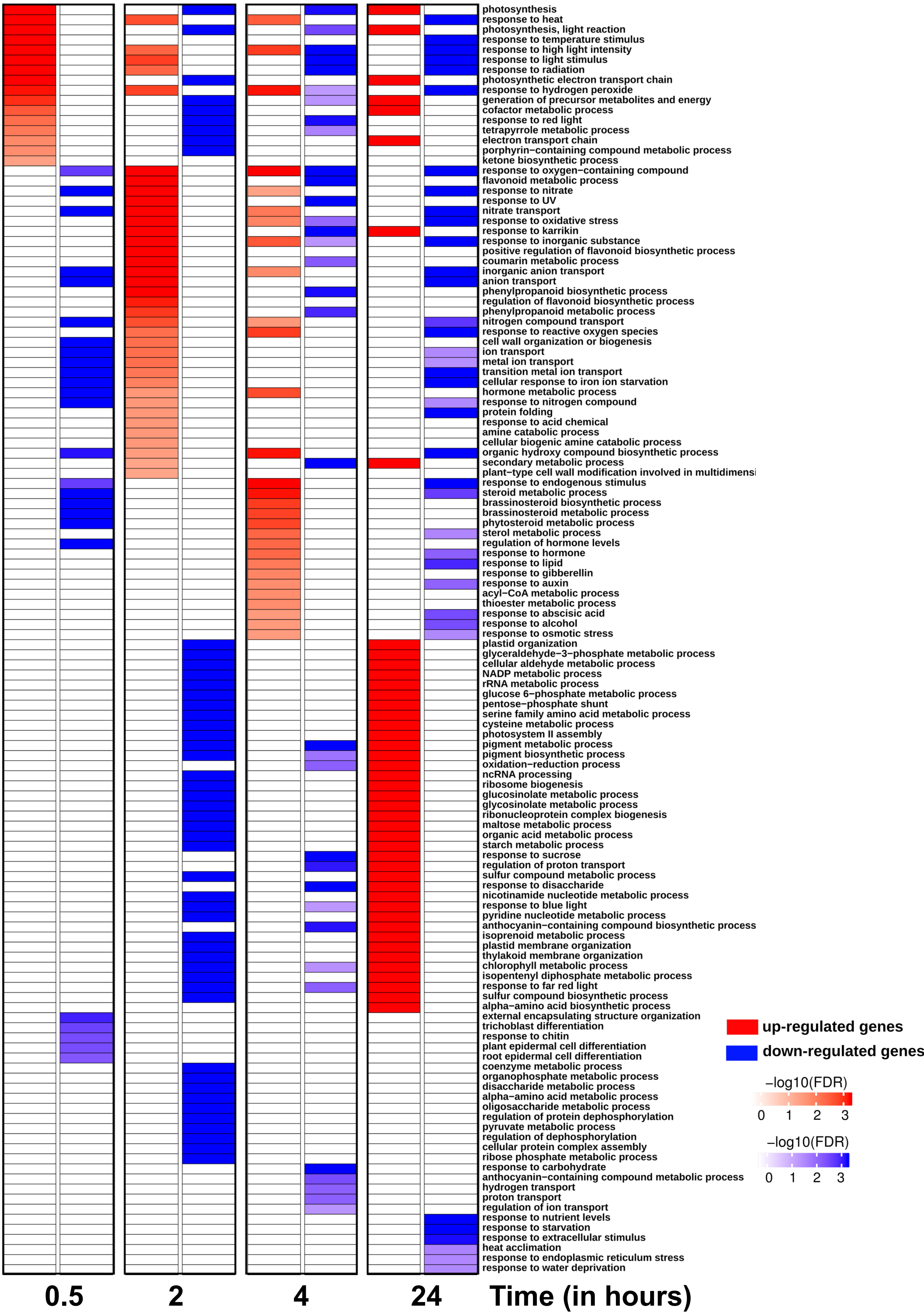

fig. S2: Gene Ontology (GO) term enrichment analysis for differentially expressed genes compared with the previous time point. Significantly enriched GO terms were identified using AgriGov2 with a custom *G. gynandra* background built by mapping *G. gynandra* proteins to their closest match in *A. thaliana* and inheriting terms from the TAIR10 annotation. Values plotted are -log10(FDR) and those associated with up-regulated and down-regulated gene sets are shown in red and blue respectively. Light and photosynthesis related terms were enriched in genes upregulated at 0.5 hours. Primary and secondary metabolism terms were enriched in genes upregulated at 24 hours of light suggesting that photosynthates are being produced by the end of the time-course.

fig. S3

DNaseI-SEQ

DNaseI-SEQ Reads

Sample	NMAP (Number of mapped reads, with a MAPQ score >42)	Signal Portion of Tags Fraction of tags that fall into a DHS from a sample of 5 million reads (SPOT > 0.25)
0H	240347172	0.68
0.5H	207680388	0.32
2H	211382604	0.28
4H	240288496	0.38
24H	245832318	0.36

DNase Hypersensitive Site (DHSs)

Sample	No. of DHS	Mean DHS (in bp)
0H	249956	649
0.5H	136187	533
2H	96478	571
4H	140017	565
24H	145970	675

Deproteinized genomic DNA extraction from different de-etiolating time course samples

↓

Bias is calculated for each time point by dividing frequency of cuts for each 6-mer by background frequency

↓

6-mer	6-mer freq. in genome	DNaseI cut freq.	Cutting bias
AAAAAA	0.05	0.01	2
AAAAAC	0.06	0.05	0.83
AAAAAG	0.05	0.04	2
-	-	-	-
-	-	-	-
TTTTTT	n	n	n

↓

FootPrintMixture.R incorporates bias file to correct DGFs for DNase-I cutting bias

↓

DGF with Footprint Likelihood Ratios (FLR) <0 are filtered out

↓

Digital Genomic Foot-printing after filtering

Sample	Initial no. DGF	No. DGFs post bias correction
0h	99135	87974
0.5h	27186	24727
2h	37989	34367
4h	17386	12736
24h	81713	73998

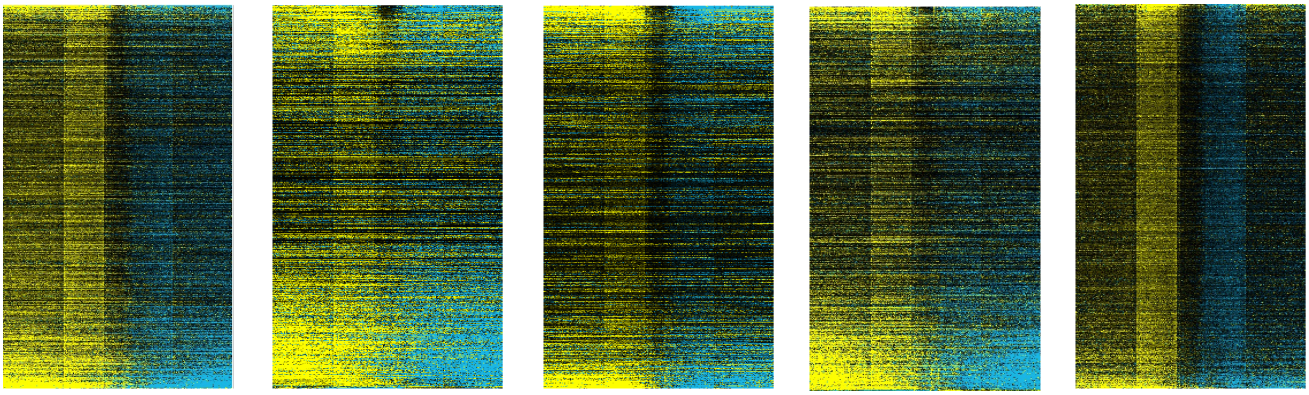

Time (hours) 0 0.5 2 4 24

fig. S3: Pipeline for DNaseI-SEQ data processing. On the top left-hand side, pooled reads passed through quality control before DHS identification using MACS2 peak-calling. The DHSs, representing accessible chromatin regions, were then searched for DGF using the pyDNase package. These DGF were prone to distorting effects due to DNaseI bias in DNA digestion. On the top right, the pipeline for identifying this bias is shown which includes the DNaseI digestion of deproteinised ("naked") gDNA. This generates 6-mer frequencies at each cut site which is used as input for the FootPrintMixture.R tool that scores the Footprint Likelihood Ratio (FLR) of each DGF (likelihood of being a true positive). DGF with FLR < 0 were removed leaving a final set of DGF used for analysis. Heatmap of cut patterns shown centred around each DGF.

fig. S4

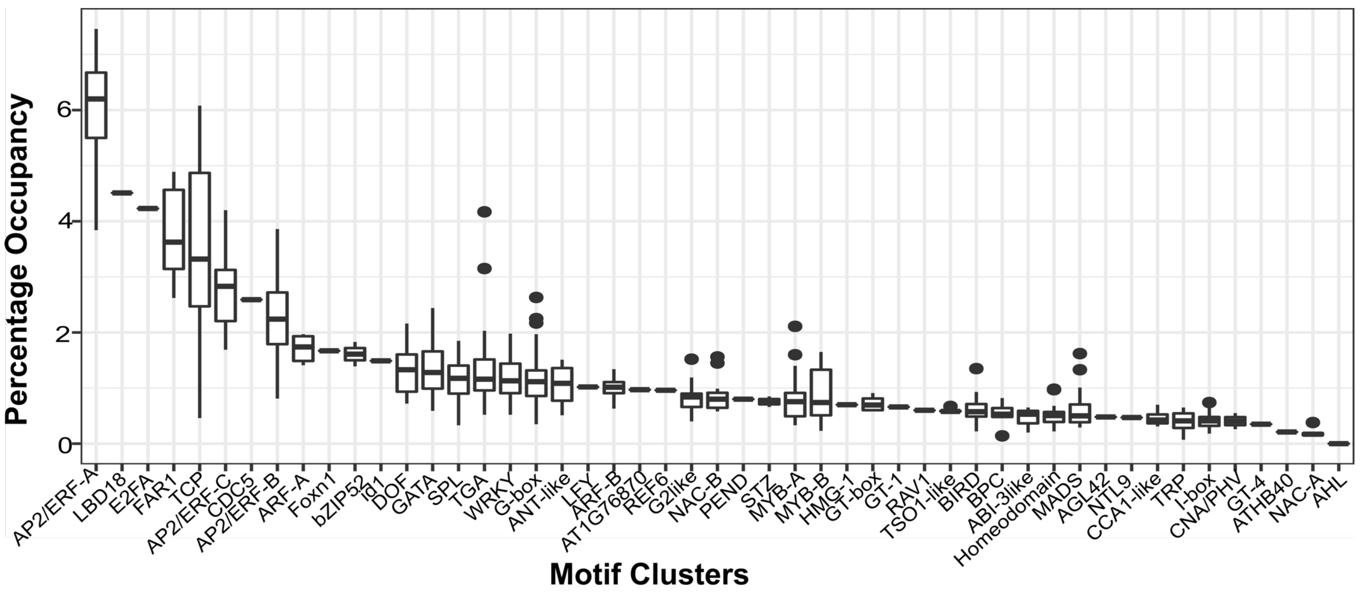

fig. S4: Percentage occupancy represents the proportion of potential binding sites identified by scanning DHS regions for motifs that are entirely contained within a DGF. Data are grouped by motif cluster. Higher occupancy could represent greater activity, abundance or binding affinity.

fig. S5

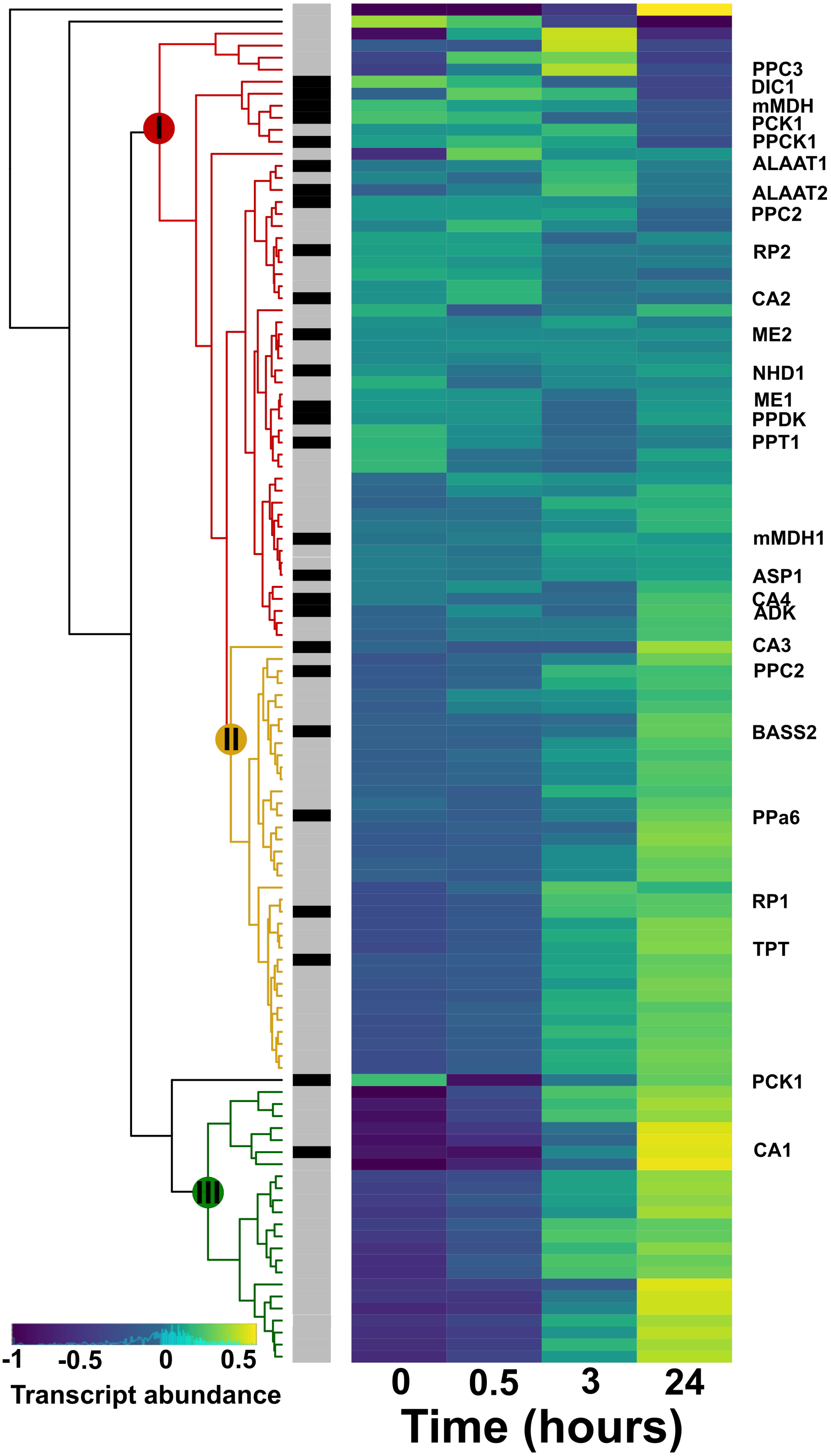

fig. S5: Expression patterns of photosynthesis genes (grey sidebar) and C_4 genes (black sidebar) in *A. thaliana* during de-etiolation. Heatmap illustrating transcript abundance with each gene being represented by a row, and data centred around the row mean. Dendrograms (red, yellow and green) highlight distinct expression clusters representing strong, moderate or no induction respectively.

fig. S6

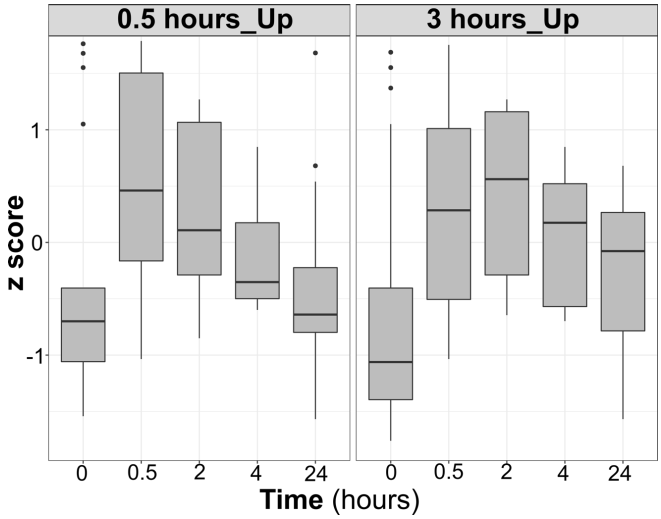

fig. S6: Expression profiles (TPM normalised z score) of *G. gynandra* orthologs (n = 17 for each group) for the most strongly induced transcription factors in *A. thaliana* at 0.5 versus 0 hours (n = 11) and 3 versus 0 hours (n = 13). *G. gynandra* transcription factors show strong induction to light, indicating conservation in induction dynamics across these two species.

fig. S7

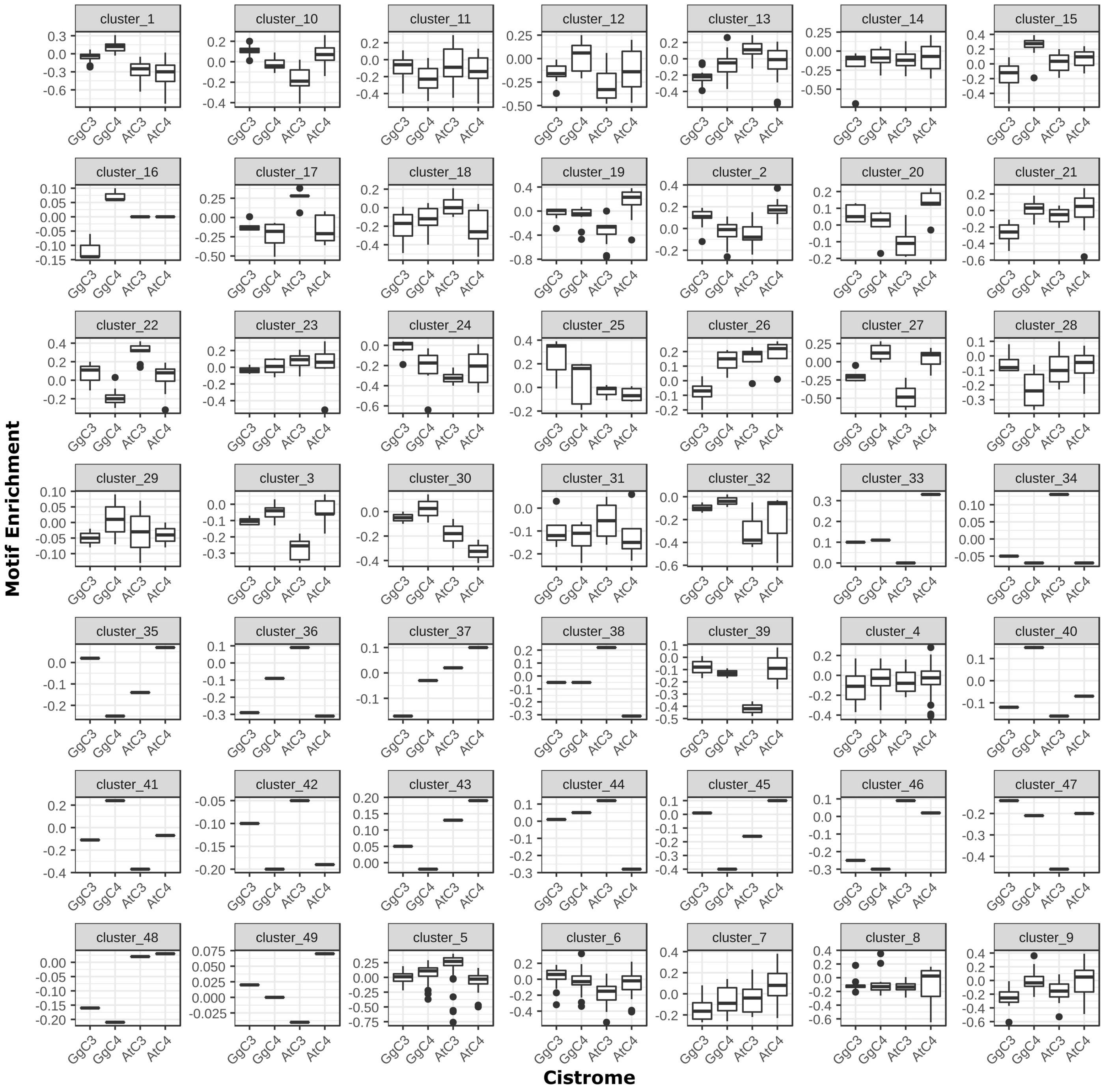

fig. S7: Motif enrichment grouped by motif clusters. Enrichment represented as log fold ratio of observed frequency of motif in the cistrome compared with the expected frequency calculated from the background.

fig. S8

AtPPDKpro::GUS

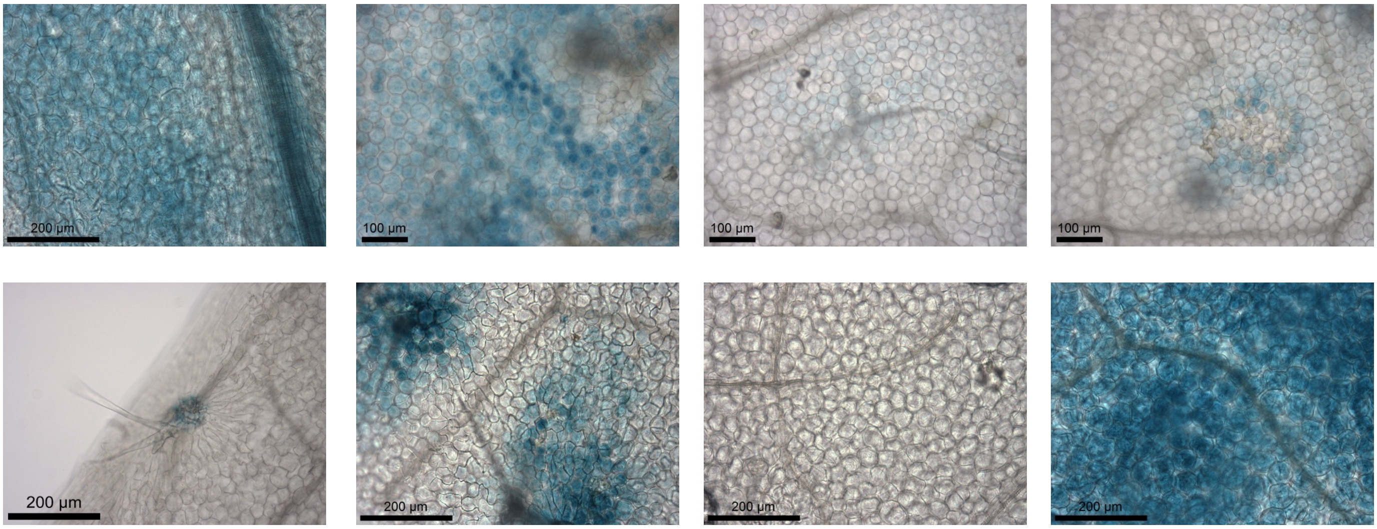

GgPPDKpro::GUS

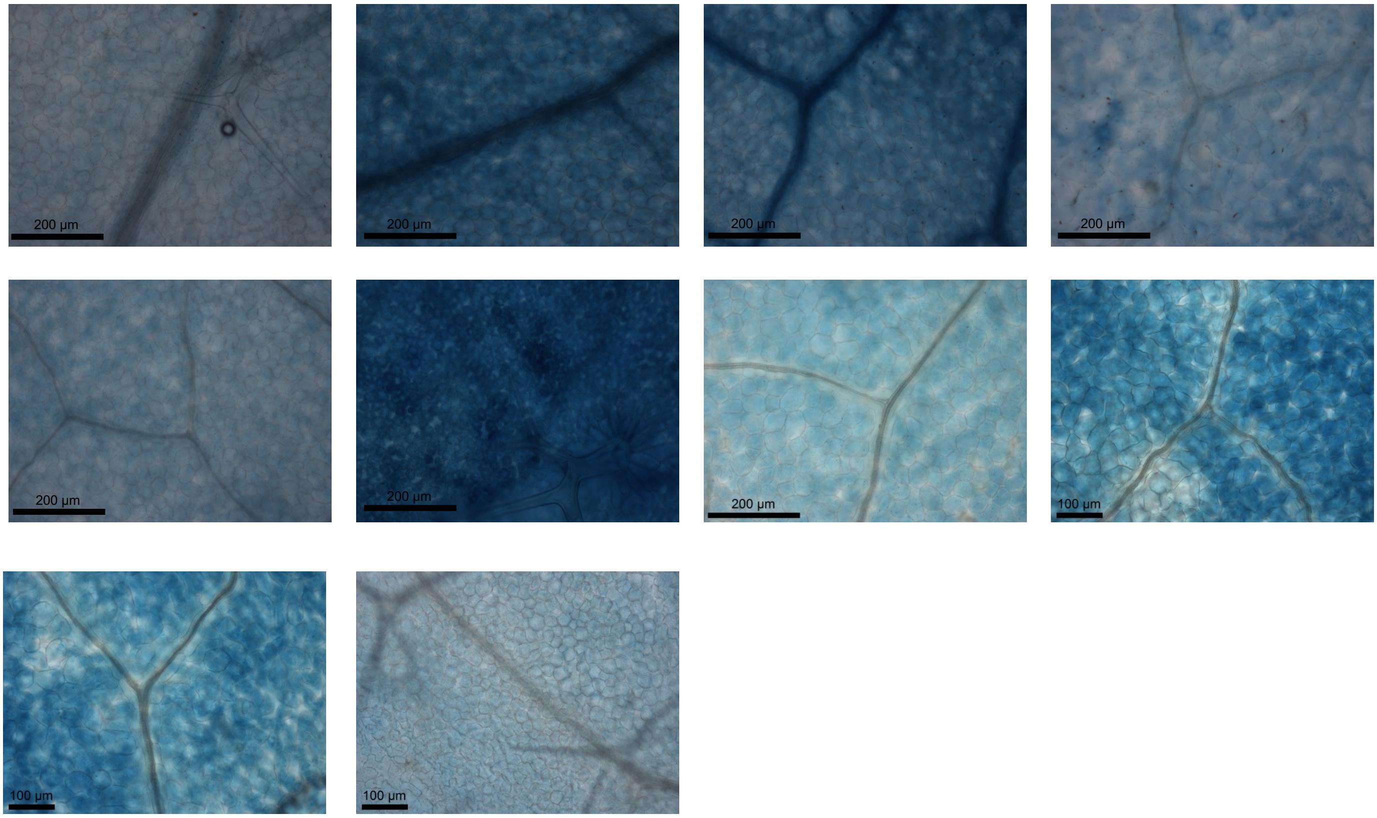

fig. S8: Representative images from eight and ten independent T1 lines of *A. thaliana* containing promoter::GUS fusions of the *PPDK* gene from *A. thaliana* (*proAtPPDK*) or *G. gynandra* (*proGgPPDK*) respectively. Staining performed for 18 hours. Scale bars represent 100 or 200 µm.

fig. S9

AtASP1pro::GUS

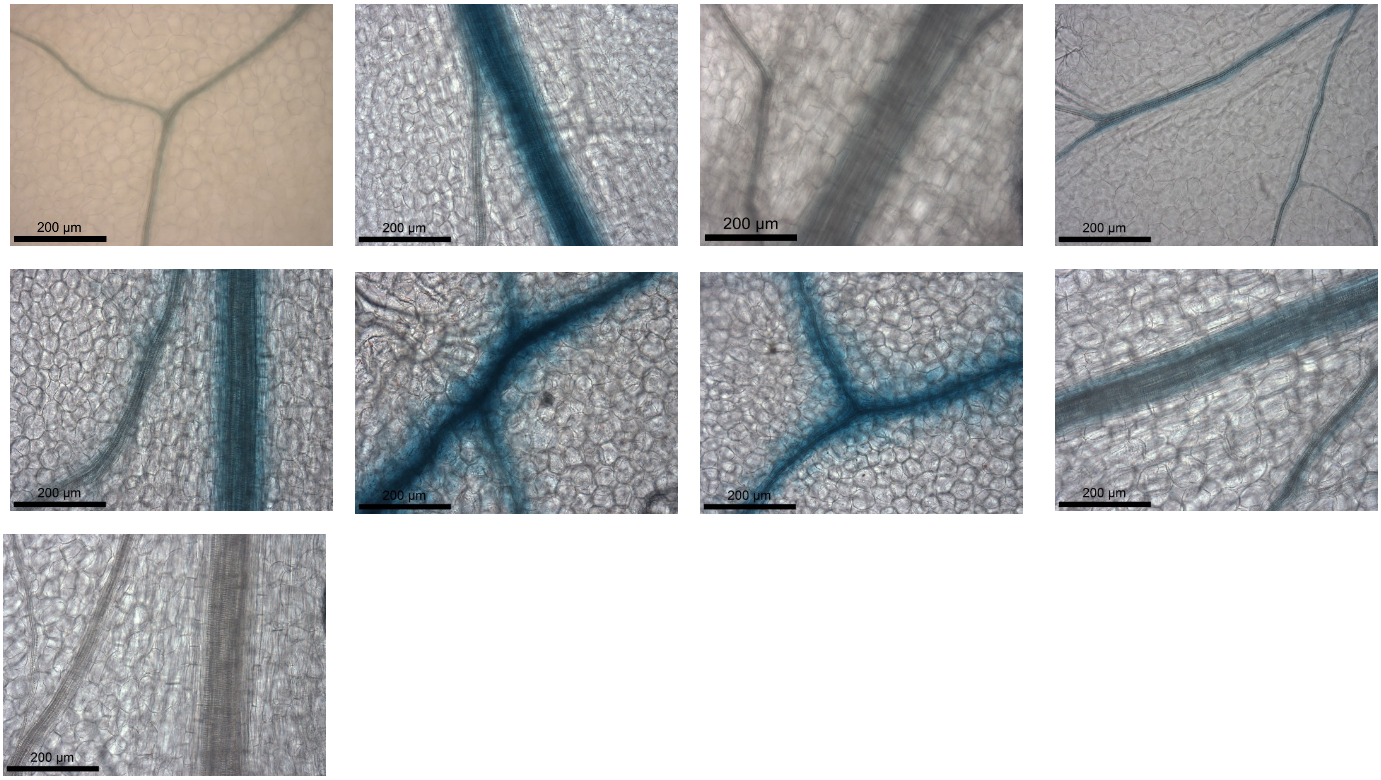

GgASP1pro::GUS

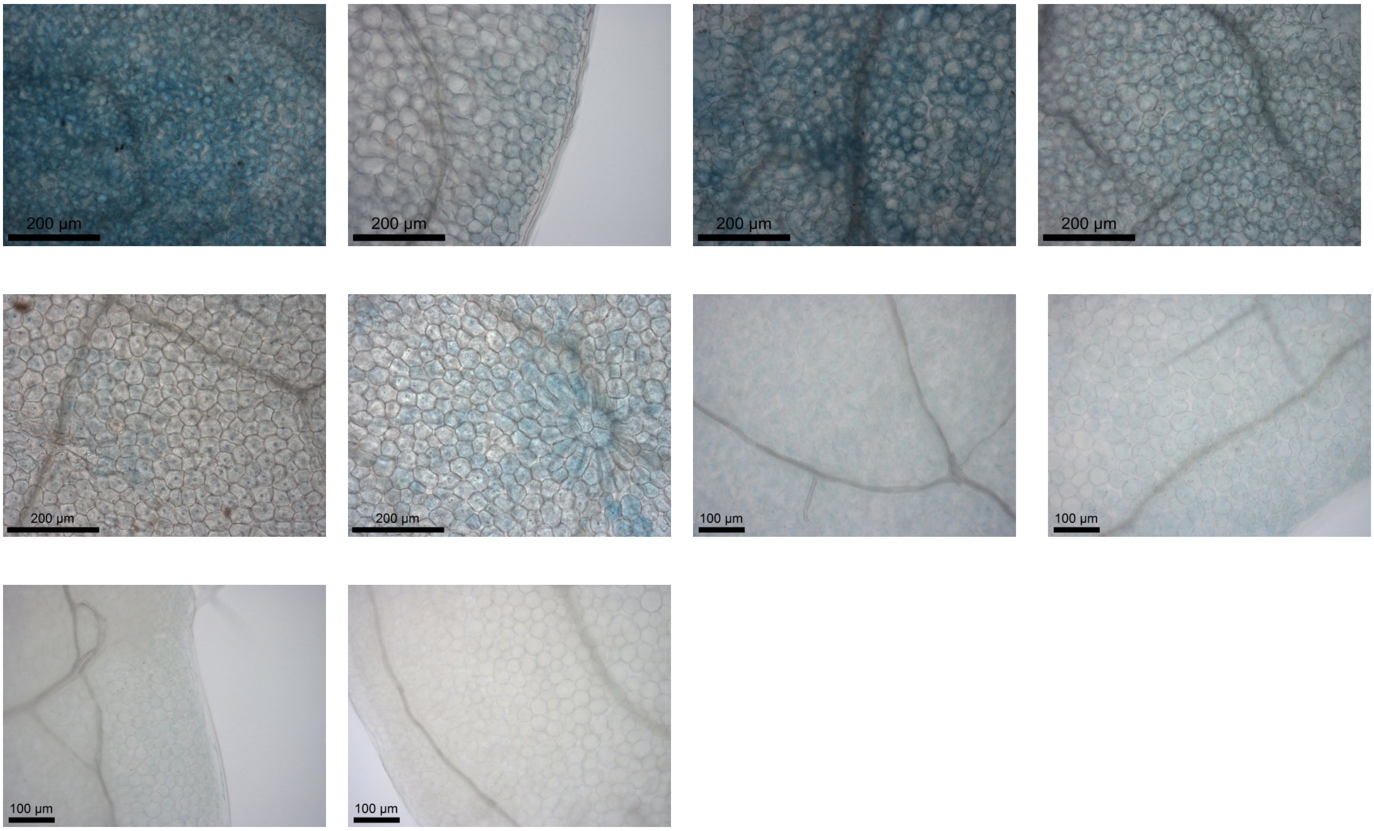

fig. S9: Representative images from nine and ten independent T1 lines of *A. thaliana* containing promoter::GUS fusions of the *ASP1* gene from *A. thaliana* (*proAtASP1*) or *G. gynandra* (*proGgASP1*) respectively. Staining performed for 18 hours. Scale bars represent 100 or 200 µm.

fig. S10

AtPPa6pro::GUS

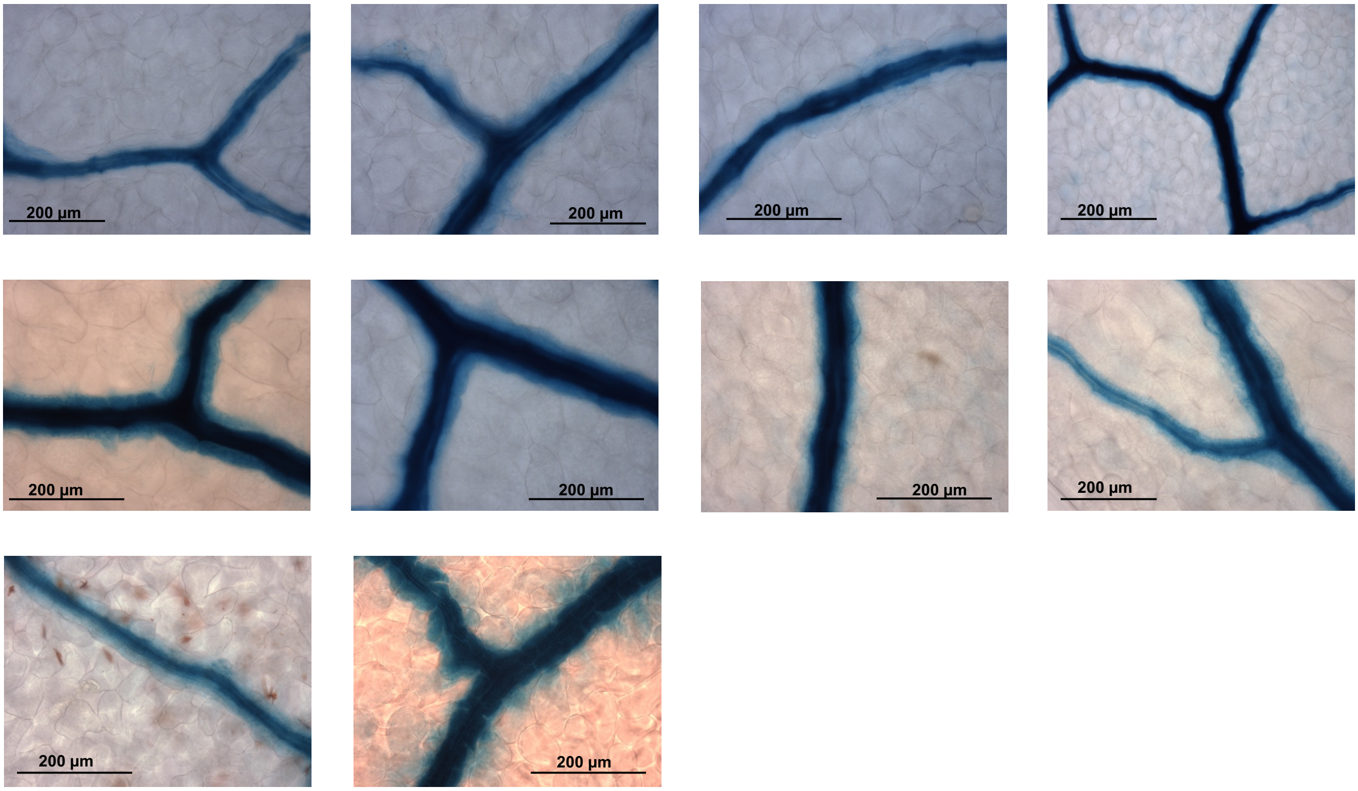

GgPPa6pro::GUS

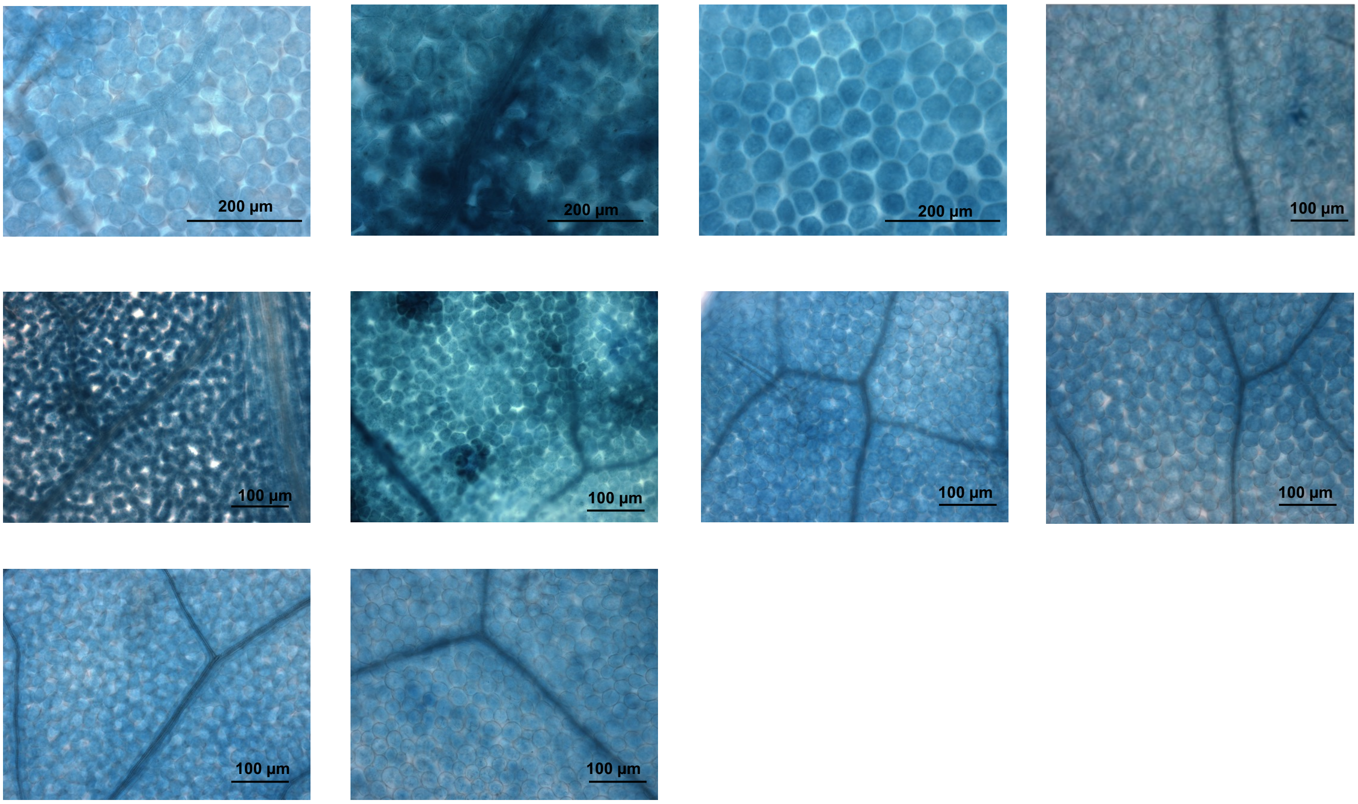

fig. S10: Representative images from ten independent T1 lines of *A. thaliana* containing promoter::GUS fusions of the *PPa6* gene from *A. thaliana* (*proAtPPA6*) or *G. gynandra* (*proGgPPA6*). Staining performed for 18 hours. Scale bars represent 100 or 200 µm.

fig. S11

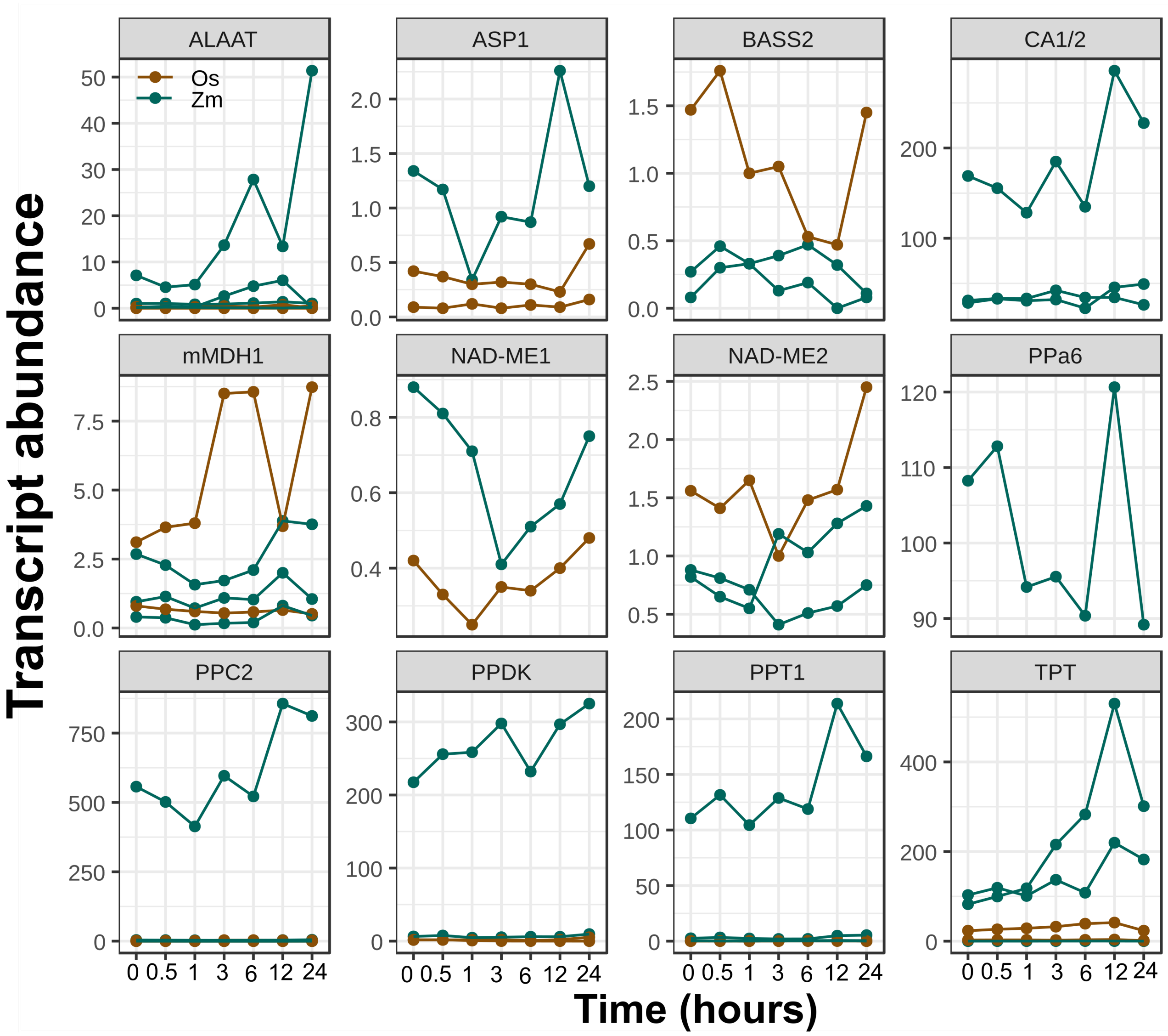

fig. S11: Line plots showing mean normalised expression values at each time point for both *O. sativa* and *Z. mays* across their respective de-etiolation time-courses. Data from this monocot de-etiolation study [47] was processed in the same way as that described for *G. gynandra* and *A. thaliana*. Many orthogroups showed similar patterns to those defined in *G. gynandra* and *A. thaliana* such that one or more C_4 paralog(s) from *Z. mays* showed much higher abundance than other members of the orthogroup.

fig. S12

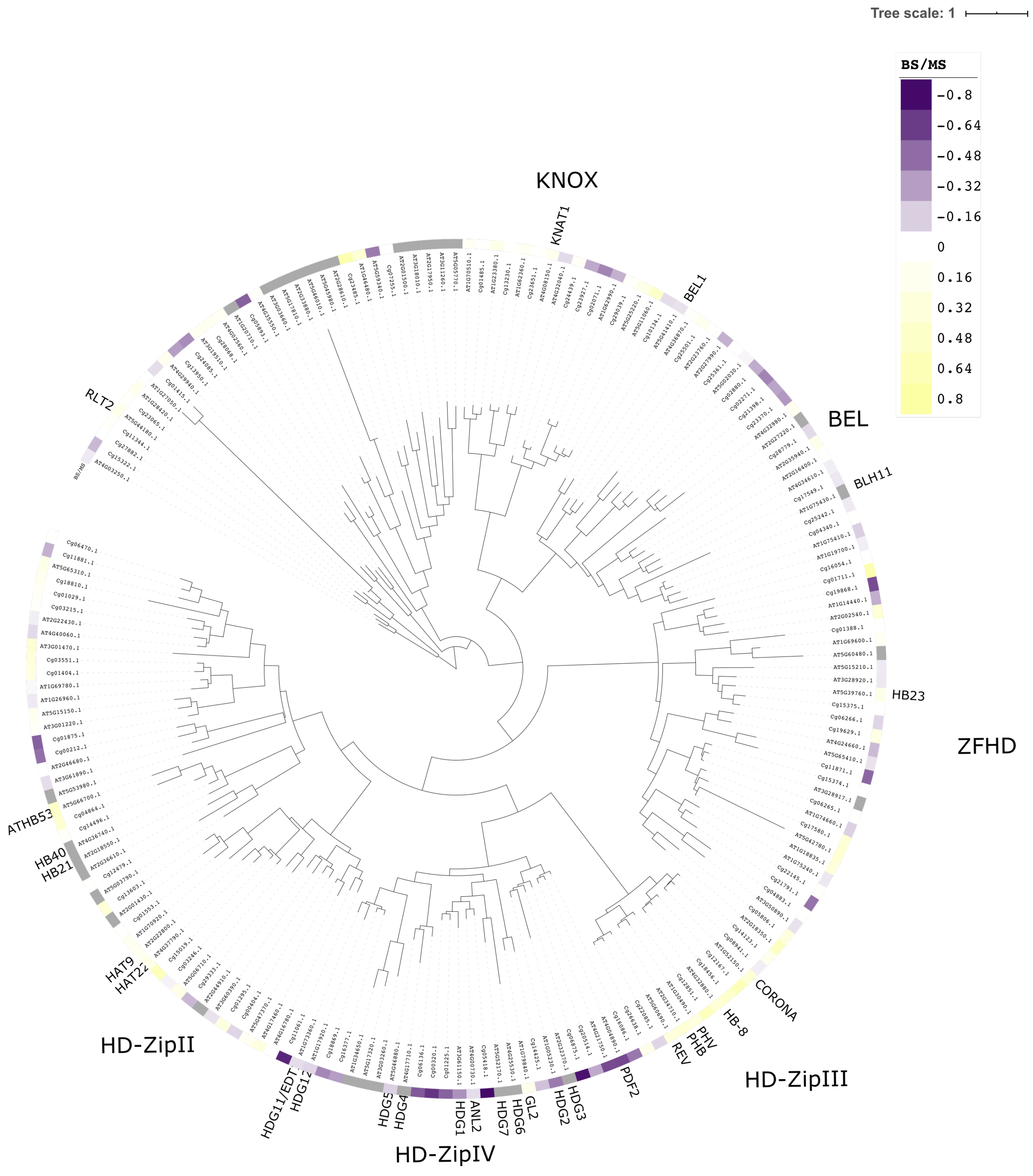

fig. S12: Unrooted phylogenetic tree of homeodomain protein sequences taken from *G. gynandra* and *A. thaliana*. Analysis was carried out using the ete3 pipeline and visualised with the iTOL web tool. Where data were available, each leaf is annotated with the ratio of bundle sheath to mesophyll expression from publicly available datasets [62, 63]. The HD-Zip IV family is preferentially expressed in the mesophyll in both species in contrast to the HD-Zip III family. Major family names and individual gene names of interest shown.

ONLINE SUPPLEMENTARY MATERIALS
Available on Dryad https://datadryad.org/stash/dataset/doi:10.5061/dryad.sf7m0cgb2

SUPPLEMENTARY TABLES:

Captions for Table S1:
Sequencing and mapping statistics for three biological replicates of different time course treatments of de-etiolating *G. gynandra* seedling.

Captions for Table S2:
List of C_4 photosynthesis and Photosynthesis genes.

Captions for Table S3:
Transcription factors found among the differentially regulated genes at each time point. Top blastp hits to Arabidopsis are shown.

Captions for Table S4:
Summary statistics of the DHS called on time-point replicates. The two replicates used were the deep sequenced sample and the replicate made from pooling the two shallow replicates. One of the shallow four-hour replicates failed QC and so only one was used. DHS overlap between the two replicates was compared to a randomly shuffled overlap with the real overlaps showing between ~1.9 and 3 times the random overlap.

Captions for Table S5:
Plant Jaspar Motifs were clustered using the RSAT motif clustering tool into 49 motif groups. Many, but not all motif groups were made up of motifs recognised by members of the same transcription factor family. Not all transcription factor family members' motifs were in the same motif cluster.

Captions for Table S6:
Statistical analysis with p-values for chromatin accessibility changes between 0 vs 24 hours of lights in regions highlighted in boxes in Fig.5C. The read depth values within each box region were compared between the 0- and 24-hours light.

Captions for Table S7:
Arabidopsis orthologs to *G. gynandra* transcription factors found to show strong light responsive gene induction. Arabidopsis orthologs show conservation in light response profile.

Captions for Table S8:
List of 582 genes showing strong induction on light.
